# Structural and evolutionary analyses of the mitochondrial genome of *Spuriopimpinella brachycarpa*

**DOI:** 10.3389/fpls.2024.1492723

**Published:** 2024-11-26

**Authors:** Jun Han, Wenbo Xu, Huanxi Yu, Yun Han, Ming Zhu

**Affiliations:** ^1^ Chinese Medicine Research Institute of Beijing Tcmages Pharmaceutical Co., Ltd., Beijing, China; ^2^ Nanjing Institute of Environmental Sciences, Ministry of Ecology and Environment of the People’s Republic of China, Nanjing, China; ^3^ College of Life Sciences, South China Agricultural University, Guangzhou, China

**Keywords:** *Spuriopimpinella*, *Pimpinella*, mitochondrial genome, phylogenetic analysis, RNA editing, chloroplast-mitochondrial homologous fragments

## Abstract

**Introduction:**

*Spuriopimpinella brachycarpa* (Kom.) Kitag., a member of the Apiaceae family, is a perennial aromatic herb native to Northeast Asia with applications in culinary and traditional medicine. Despite its significance, most studies on *S. brachycarpa* have primarily focused on its phytochemical properties, with limited insights into its molecular and genomic characteristics.

**Methods:**

This study presents the sequencing and assembly of the mitochondrial genome (mitogenome) of *S. brachycarpa* using second- and third-generation high-throughput sequencing technologies. Comprehensive analyses were performed on its structural organization, RNA editing sites, relative synonymous codon usage (RSCU), and repeat sequences. Comparative analyses with closely related species were also conducted.

**Results:**

The mitogenome exhibited a multi-branched structure, with a total length of 523,512 bp and a GC content of 43.37%. Annotation revealed 30 unique protein-coding genes, 21 tRNA genes, and three rRNA genes. Comparative analysis indicated that the *S. brachycarpa* mitogenome contains structural variations but shares collinear features with other Apiaceae species. We identified 618 potential RNA editing sites involving C-to-U conversions and discovered 59 homologous fragments between the mitogenome and plastome, comprising 8.13% of the mitogenome.

**Discussion:**

These results enrich the genomic database of Apiaceae, providing valuable insights into the evolutionary relationships and genetic diversity within the family.

## 1 Introduction

The Apiaceae (syn. Umbelliferae) family, known for its abundant species and remarkable morphological diversity, consists of 466 recognized genera and roughly 3,800 species (Plunkett et al., 2018). Members of this family range from small herbs to trees and are predominantly found in temperate regions, with a significant presence in Central Asia (Pimenov and Leonov, 1993; Sheh et al., 2005). Additionally, Apiaceae holds significant economic value, serving various medicinal, culinary, and spice purposes (Clarkson et al., 2021), including well-known species such as carrots (*Daucus carota*), coriander (*Coriandrum sativum*), and cumin (*Cuminum cyminum*). The genus *Spuriopimpinella* was first established by Kitagawa (Kitagawa, 1941), having initially been part of *Pimpinella*, one of the largest genera in the subfamily Apioideae (Boissieu, 1906; Pimenov and Leonov, 1993). Based on molecular and morphological evidence, *Spuriopimpinella* has been described as an independent lineage distinct from *Pimpinella* and is now widely accepted (Downie et al., 2010; Wang et al., 2013). *Spuriopimpinella* is a small genus, with six accepted species, mainly distributed across East Asia, particularly in China, the Korean Peninsula, and Japan (Govaerts et al., 2021). *Spuriopimpinella brachycarpa* (Kom.) Kitag., a perennial aromatic herb, is native to Northeast Asia. Based on morphological and cytological similarities, Wang et al. reinstated *S. brachycarpa* from *Pimpinella*, supported by molecular evidence from ITS and plastid intron sequences. Molecular phylogenetic analyses also led to the reclassification of *Pimpinella arguta* into *Spuriopimpinella*, implying that molecular evidence plays an important role in taxonomic research of this genus (Wang et al., 2013). Additionally, *S. brachycarpa* holds economic significance, serving as both a culinary herb and a traditional medicine. For instance, the plant contains flavonoids, alkaloids, and phenolic compounds that can be used to treat colds, coughs, indigestion, and abdominal pain (Wu et al., 2023). The leaves and stems of *S. brachycarpa* are consumed as vegetables and seasonings (Zheng, 2016). Despite its economic and medicinal significance, most research on *S. brachycarpa* has been limited to analyzing its phytochemical properties with little exploration at the molecular level, particularly in the genome.

Mitochondria are thought to have originated from a primordial endosymbiotic event and now play a vital role in plant cells by acting as the primary sites for aerobic respiration (Margulis, 1970; Gray et al., 1999; Wallace, 1999; Dyall et al., 2004; Poole and Penny, 2007; Koonin, 2010). Mitochondria facilitate the oxidation of saccharides, fats, and amino acids to release energy necessary for cellular activities (Nicholls and Budd, 2000; Houten and Auwerx, 2004). In addition to energy production, mitochondria also contribute to cell differentiation, signal transduction, apoptosis, growth, and cell cycle regulation (Scorrano et al., 2002; Mannella, 2006). Plant mitogenomes are notably diverse, reflecting lineage-specific evolutionary processes, and differ significantly from their animal counterparts in terms of size and structure (Chen et al., 2017). Although the mitochondrial genome (mitogenome) is typically represented as a circular double-stranded structure, it can also include multiple independent chromosomes and linear or multi-branched structures (Gualberto and Newton, 2017; Kozik et al., 2019). Generally, plant mitochondria tend to incorporate foreign DNA, leading to a large number of repeats that often serve as sites of genomic recombination, thereby contributing to their structural complexity (Jiang et al., 2023; Li et al., 2024). Compared to plastomes, the structural complexity of mitogenomes poses significant challenges for sequencing and assembly (Arrieta-Montiel and Mackenzie, 2011; Štorchová and Krüger, 2024). Recent advancements in long-read sequencing technologies, such as PacBio and Oxford Nanopore, have made the accurate assembly of complex plant mitogenomes feasible, overcoming limitations posed by traditional short-read sequencing (Karin et al., 2023). These technological improvements have enhanced our understanding of the structural intricacies and functions of plant mitogenomes. Despite the growing body of research on plant mitogenomes, studies remain limited in certain plant families, including the Apiaceae family. To date, the NCBI database contains approximately 2,500 mitogenomes, but only 17 are from the Apiaceae family. This reveals a significant gap in genomic data, highlighting the need for further research in this area.

In this study, we sequenced the mitogenome of *S. brachycarpa* and revealed that it is composed of five circular chromosomes. We then conducted comprehensive analyses of its structure, RNA editing sites, relative synonymous codon usage (RSCU), and repeats and compared these features with those of closely related species. Examining these features, we aimed to reveal specific evolutionary patterns and structural variations that contribute to the unique properties of *S. brachycarpa*. Our study enhances the understanding of genetic diversity and evolutionary dynamics within the *Spuriopimpinella* genus and the broader Apiaceae family. Moreover, the findings offer valuable insights into the potential functional significance of *S. brachycarpa*’s mitogenome, particularly concerning its medicinal and economic applications.

## 2 Materials and methods

### 2.1 Sampling, DNA & RNA extraction, and sequencing

Samples of *S. brachycarpa* were collected from Liaoning Province, China (42.525°N, 124.148°E; **Supplementary Figure S1**). These samples were deposited at the Chinese Medicine Research Institute of Beijing Tcmages Pharmaceutical Co., Ltd. (Beijing, China) with voucher specimen SB01. Total DNA was extracted using a modified CTAB method, and RNA was extracted using a BioTeke RNA extraction kit (Raimundo et al., 2018). The high-quality extracted DNA and RNA samples were then sent to Wuhan Benagene Technology Co., Ltd. for Illumina and Oxford Nanopore Technologies (ONT) genome sequencing.

### 2.2 Genome assembly and annotation

The mitogenome contigs of *S. brachycarpa* were assembled with Flye using long-read sequences (Kolmogorov et al., 2019). Long- and short-read sequences were aligned to the contigs using BWA (Li and Durbin, 2009). Unicycler was used to assemble the aligned reads into a complete mitogenome (Wick et al., 2017), which was visualized and exported with Bandage (Wick et al., 2015). The mitogenome was annotated for protein-coding genes, tRNAs, and rRNAs using IPMGA, tRNAscan-SE, and BLASTn, respectively (Lowe and Eddy, 1997; Johnson et al., 2008). After manual correction in Apollo, the annotation files were submitted to NCBI with accession numbers PQ273107 to PQ273111 (Dunn et al., 2019). Additionally, the plastome of *S. brachycarpa* was assembled and annotated using GetOrganelle and CPGAVAS2 (Shi et al., 2019; Jin et al., 2020), with the corrected annotation uploaded to NCBI under accession number PQ213365.

### 2.3 Intraspecific mitogenome analysis

The relative codon usage of protein-coding sequences in the mitogenome was extracted and analyzed using CPStools (Huang et al., 2024a). Additionally, simple sequence repeats (SSRs) in the mitogenome were identified using CPStools with specific parameters: a minimum of 10 repeats for mononucleotides, 5 for dinucleotides, 4 for trinucleotides, and 3 for tetranucleotides, pentanucleotides, and hexanucleotides (Huang et al., 2024a). Tandem and long sequence repeats (LSRs) were detected using TRF and REPuter, with TRF configured for match, mismatch, and indel weights of 2, 7, and 7, detection parameters including a matching probability of 80% and an indel probability of 10%, a minimum alignment score of 50, and a maximum period size of 500, while REPuter was set with a Hamming distance of 3 and a minimum repeat size of 30 bp (Benson, 1999; Kurtz et al., 2001). After quality control and adapter sequence removal, transcriptome data were aligned to the assembled mitogenome, and RNA editing sites were detected with Bcftools, applying a filter to exclude variants with QUAL < 20 and depth < 10 (Danecek and McCarthy, 2017; Ou et al., 2024).

### 2.4 Comparative mitogenome analysis

Homologous fragments of mitochondrial plastid DNA segments (MTPTs) between the mitogenome and plastome were compared using BLASTn to identify regions of similarity and potential horizontal gene transfer events, with an e-value threshold of 1e-5 and a similarity of at least 70%. The results were then visualized using Circos (Krzywinski et al., 2009). Mitogenome sequences from four closely related species were downloaded from NCBI, and conserved homologous sequences over 500 bp across these species were identified using BLASTn. These conserved collinear blocks were then visualized by MCScanX (Wang et al., 2012). RNA editing sites in related species without transcriptome support were predicted using DeepRed-Mt (Edera et al., 2021). Common protein-coding genes were extracted with CPStools and aligned using MAFFT (Katoh and Standley, 2013). Phylogenetic trees were constructed with RAxML using the maximum likelihood method and 1000 bootstrap replicates (Stamatakis, 2014), with two Aquifoliales species (*Ilex pubescens* and *Ilex metabaptista*) as the outgroup.

## 3 Results

### 3.1 Genome assembly and annotation

**Figure 1:**
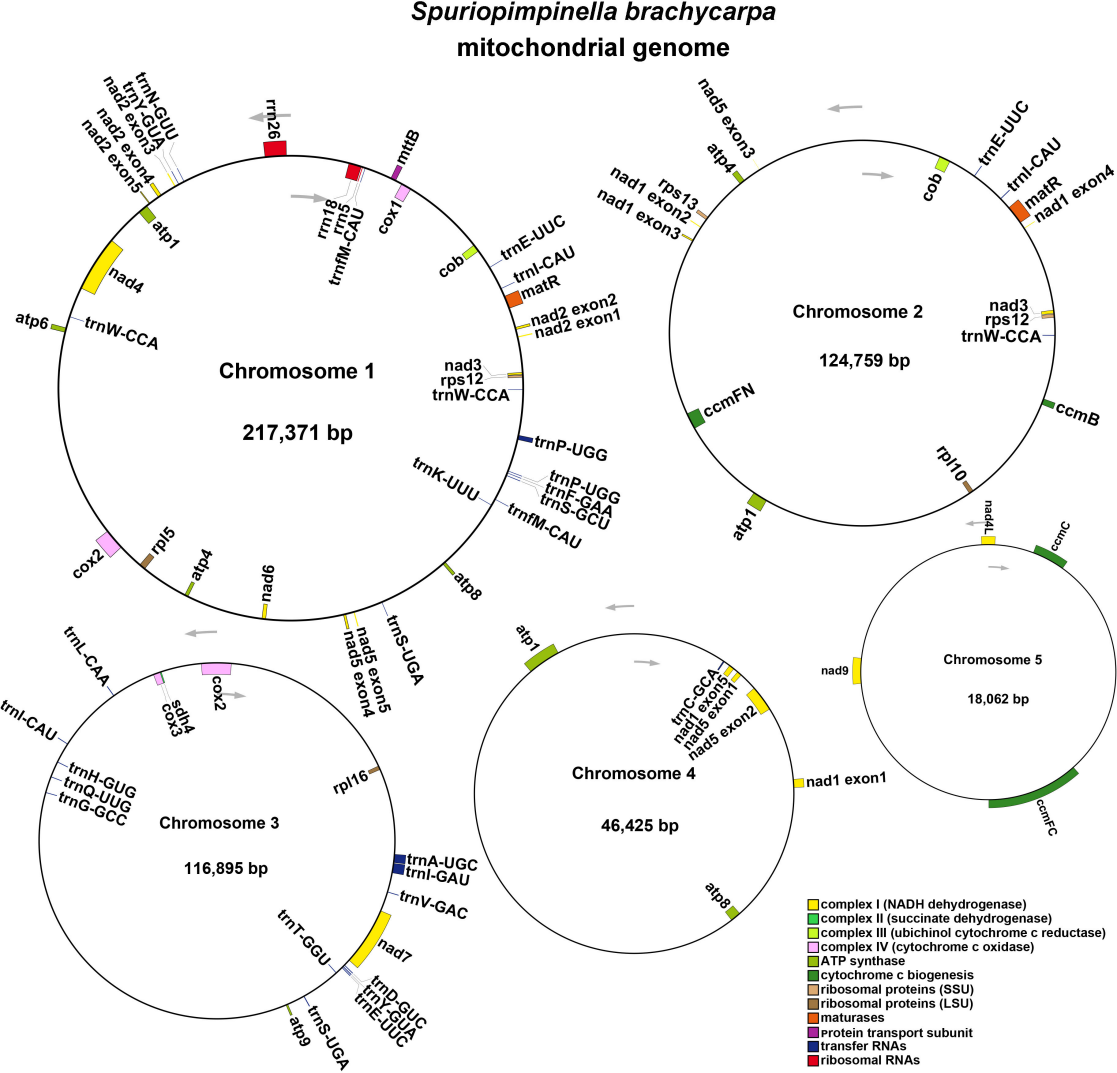
The mitogenome map of *S. brachycarpa*. Chromosomes 1-5 are indicated by five different contigs. The arrows show transcriptional direction of the mtDNA. Genes with different functions were represented using different colors.

To assemble the genome, 10.6 GB of ONT clean reads and 13.4 GB of Illumina clean reads were used. The mitogenome of *S. brachycarpa* was found to be multi-chromosomal. After excluding repeated regions from the ONT sequences, five main circular chromosomes were obtained, with a total length of 523,512 bp and a GC content of 43.37% (**Figure 1**; **Supplementary Table S1**). The lengths of the five chromosomes were 217,371 bp for chromosome 1, 124,759 bp for chromosome 2, 116,895 bp for chromosome 3, 46,425 bp for chromosome 4, and 18,062 bp for chromosome 5 (**Table 1**). The genome was annotated, revealing 30 unique protein-coding genes, including 24 core and six non-core genes, 21 tRNA genes (seven with multiple copies), and three rRNA genes (**Table 2**). The plastome of *S. brachycarpa* showed a typical tetrad structure, totaling 158,449 bp, with a GC content of 37.67% (**Supplementary Figure S2**). The large single-copy region, small single-copy region, and inverted repeats regions were 88,249 bp, 17,688 bp, and 26,256 bp, respectively, and a total of 133 genes were annotated, including 88 protein-coding genes, 37 tRNA genes, and eight rRNA genes (**Supplementary Table S2**).

**Table 1:** Type, length and depth of five chromosomes in *S. brachycarpa*.

Node	Type	Length(bp)	Depth(×)	Accession No.
Chr 1	circular	217,371	116	PQ273107
Chr 2	circular	124,759	86	PQ273108
Chr 3	circular	116,895	98	PQ273109
Chr 4	circular	46,425	104	PQ273110
Chr 5	circular	18,062	92	PQ273111

**Table 2:** Genes predicted in the mitogenome of *S. brachycarpa*.

Group of genes	Name of genes
ATP synthase	*atp*1 (×2), *atp*4 (×2), *atp*6, *atp*8 (×2), *atp*9
NADH dehydrogenase	*nad*1, *nad*2, *nad*3 (×2), *nad*4, *nad*4L, *nad*5, *nad*6, *nad*7, *nad*9
Cytochrome b	*cob* (×2)
Cytochrome c biogenesis	*ccm*B, *ccm*C, *ccm*FC, *ccm*FN
Cytochrome c oxidase	*cox*1, *cox*2 (×2), *cox*3
Maturases	*mat*R (×2)
Protein transport subunit	*mtt*B
Ribosomal protein large subunit	*rpl*5, *rpl*10, *rpl*16
Ribosomal protein small subunit	*rps*12 (×2), *rps*13
Succinate dehydrogenase	*sdh*4
Ribosome RNA	*rrn*5, *rrn*18, *rrn*26
Transfer RNA	*trn*A-UGC, *trn*C-GCA, *trn*D-GUC, *trn*E-UUC (×3), *trn*F-GAA, *trn*fM-CAU (×2), *trn*G-GCC, *trn*H-GUG, *trn*I-CAU (×3), *trn*I-GAU, *trn*K-UUU, *trn*L-CAA, *trn*N-GUU, *trn*P-UGG(×4), *trn*Q-UUG, *trn*S-GCU, *trn*S-UGA (×2), *trn*T-GGU, *trn*V-GAC, *trn*W-CCA (×3), *trn*Y-GUA (×2)

“x2”, genes with two copies; “x3”, genes with three copies; “x4”, genes with four copies.

### 3.2 RNA editing

**Figure 2:**
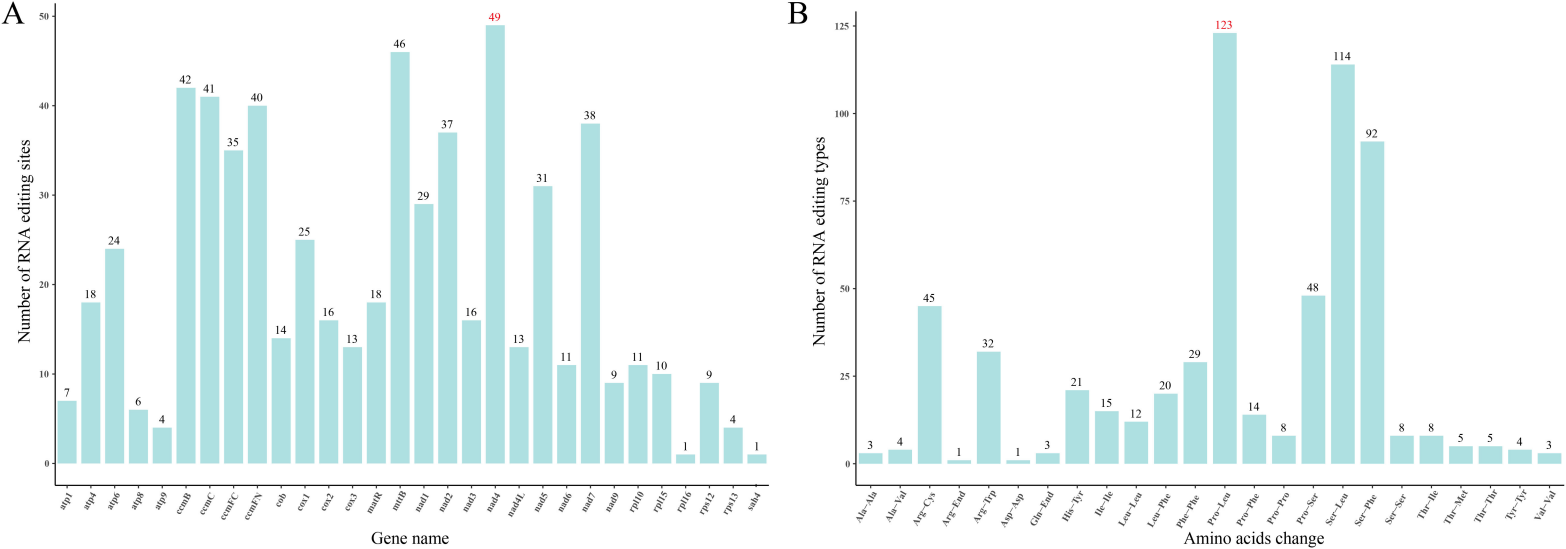
RNA editing events in *S. brachycarpa*. **(A)**, Number of RNA editing sites in each gene, **(B)**, Number of amino acid changes before and after RNA editing.

In the mitogenome of *S. brachycarpa*, RNA editing was identified across 30 unique protein-coding genes (**Figure 2A**). A total of 618 potential C-to-U RNA editing sites were detected, all of which were C-to-U conversions. The *nad*4 gene exhibited the highest number of edits, with 49 sites, followed closely by *mtt*B, which had 46 sites (**Supplementary Table S3**). Notably, RNA editing was observed at six sites within start codons (*cox*1 and *nad*4L) and stop codons (*atp*9, *mat*R, *atp*6, and *ccm*FC). The majority of the RNA editing sites were located at the first and second codon positions, with 95.63% resulting in amino acid changes. Second-position edits were particularly frequent, occurring at 62.23%. The amino acid changes showed a strong bias towards specific codon edits. For example, 123 amino acids were altered from proline (Pro) to leucine (Leu), accounting for 19.87% of the total RNA editing events (**Figure 2B**). In addition, RNA editing sites were also predicted in four related species, with 416 to 540 sites identified (**Supplementary Table S3**). Similar to *S. brachycarpa*, the two most frequent types of conversions in these species were from Pro to Leu and from serine (Ser) to Leu, representing 38.29% to 43.88% of all edits.

### 3.3 Codon preference analysis

**Figure 3:**
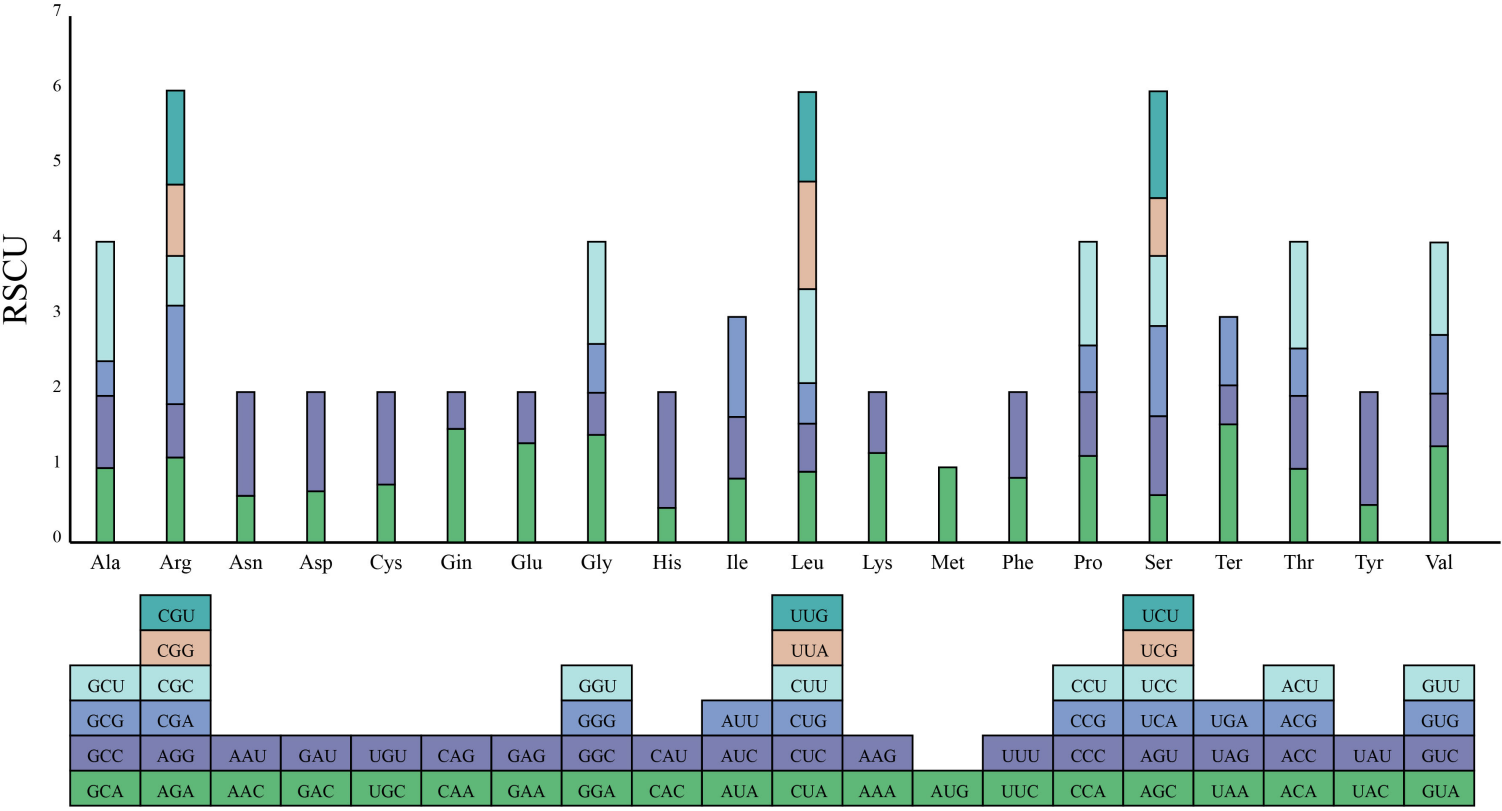
Codon usage bias of mitochondrial protein-coding genes in *S. brachycarpa*. The X-axis represents 21 amino acids, while the Y-axis shows the codons for each amino acid and their corresponding RSCU values.

In the complete mitogenome of *S. brachycarpa*, 7,679 codons from 30 unique protein-coding genes were extracted and analyzed for codon preference. Sixty-four codons encoding 21 amino acids were identified. Of these, 28 codons had RSCU values greater than 1, indicating a higher preference in the mitogenome of *S. brachycarpa* (**Figure 3**; **Supplementary Table S4**). The codon GCU (encoding alanine) showed a strong preference with an RSCU value of 1.59. Codon usage was also analyzed in four related species: *Cuminum cyminum* (12,192 codons), *Daucus carota* subsp. *sativus* (10,187 codons), *Oenanthe linearis* (8,870 codons), and *Oenanthe thomsonii* (8,730 codons) (**Supplementary Table S4**). The most preferred codon in *O. linearis* and *O. thomsonii* matched that of *S. brachycarpa*, while UAA (a stop codon) was the most preferred in *C. cyminum* and *D. carota* subsp. *sativus*.

### 3.4 Repeat analysis

**Figure 4:**
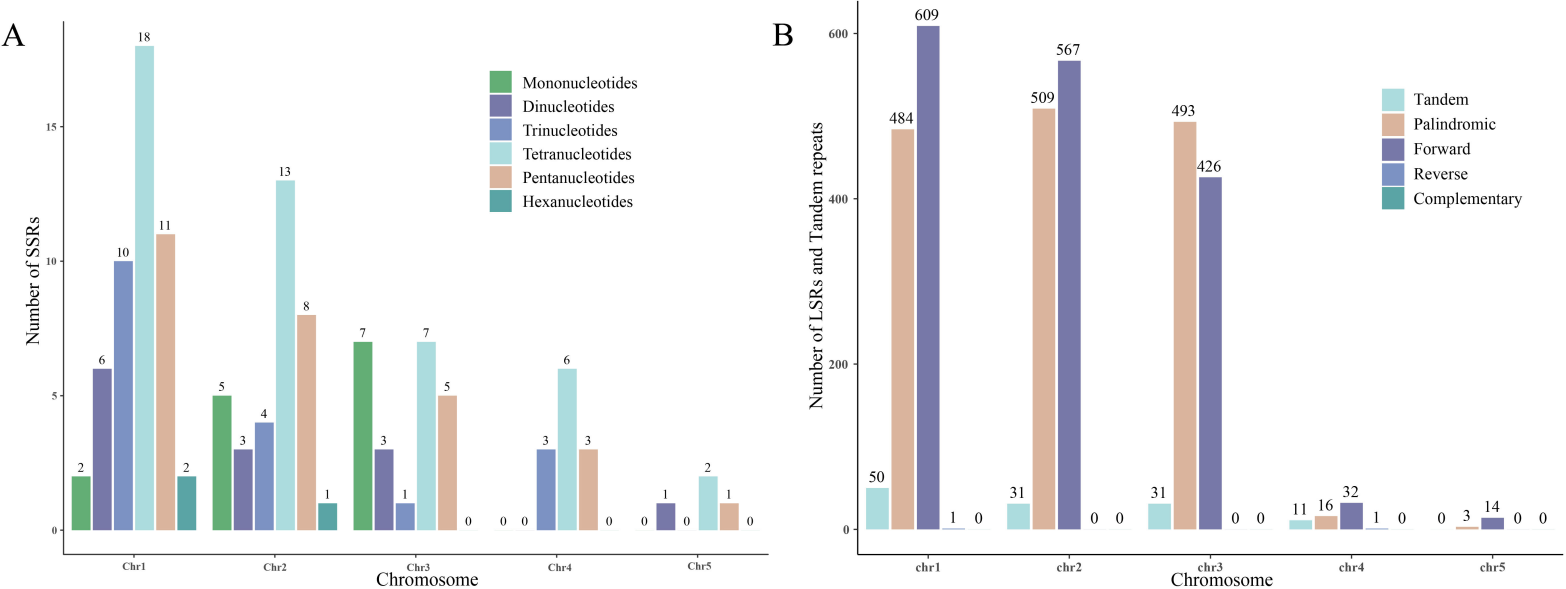
The distribution of SSRs, LSRs, and tandem repeats across the five chromosomes. **(A)**, The number of SSRs in each of the five chromosomes. **(B)**, The number of LSRs and tandem repeats in each of the five chromosomes.

In the *S. brachycarpa* mitogenome, a total of 49, 34, 23, 12, and four SSRs were identified on chromosomes 1 to 5, respectively. These SSRs exhibit varying distributions across the chromosomes, with chromosome 1 containing the highest number and chromosome 5 the least. In addition to SSRs, tandem repeats were also found in similar patterns, with counts of 50, 31, 31, 11, and 0 on chromosomes 1 to 5, respectively (**Figure 4A**). LSRs were detected in all five chromosomes, showing diverse repeat types and counts. Specifically, chromosome 1 exhibited 1,094 LSR pairs, including 484 palindromic, 609 direct, and one inverted repeat. Chromosome 2 had 1,076 LSR pairs, comprising 509 palindromic and 567 direct repeats. Chromosome 3 showed 919 LSR pairs, with 493 palindromic and 426 direct repeats. Chromosome 4 contained 49 LSR pairs, made up of 16 palindromic, 32 direct, and one inverted repeat, while chromosome 5 had 17 LSR pairs, with three palindromic and 14 direct repeats (**Figure 4B**). No complementary repeats were detected in any of the chromosomes, highlighting the absence of this repeat type in the *S. brachycarpa* mitogenome.

### 3.5 Plastome-derived mitogenomic sequence

**Figure 5:**
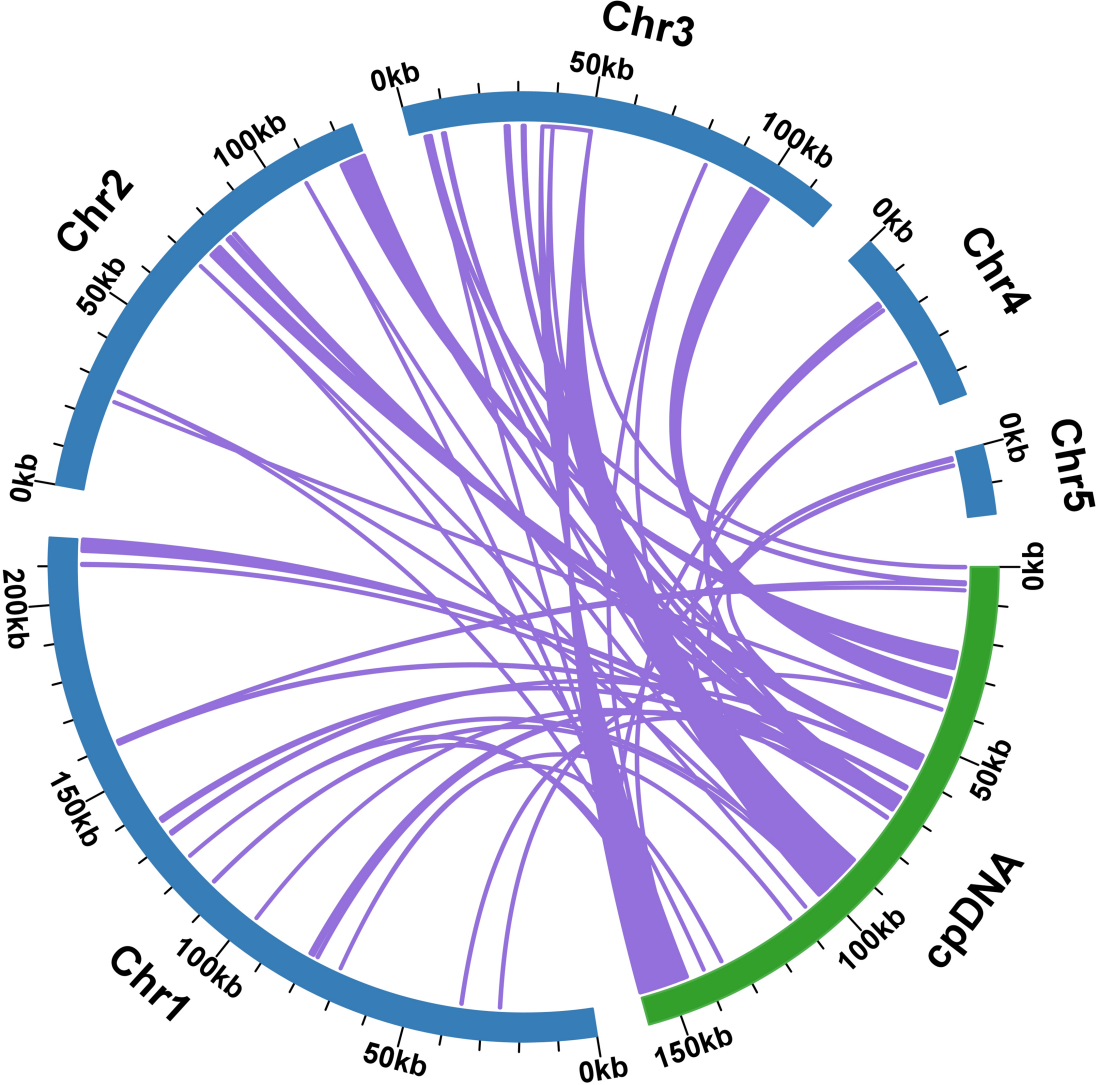
Schematic representation of MTPT (mitochondrial plastid DNA) in *S. brachycarpa*. The comparison illustrates the MTPT sequences from the mitogenome (light blue) and the plastome (light green). Arcs indicate the connections between corresponding sequence fragments between the genomes.

A total of 59 homologous fragments were identified between the mitogenome and plastome of *S. brachycarpa*, covering a combined length of 42,553 bp and representing 8.13% of the total mitogenome length (**Figure 5**). The homologous fragments varied in length, with the longest fragment, MTPT15, measuring 10,520 bp, while the shortest fragments, MTPT36 and MTPT37, were each 34 bp. Among these homologous sequences, 20 complete genes were detected, including 11 protein-coding genes and nine tRNA genes. The identified protein-coding genes comprised *atp*B, *pet*G, *pet*L, *psb*C, *psb*D, *rpl*2, *rpl*23, *rpo*B, *rps*7, *ycf*2, and *ycf*15, while the tRNA genes included *trn*D-GUC, *trn*E-UUC, *trn*H-GUG, *trnI*-CAU, *trn*L-CAA, *trn*N-GUU, *trn*T-GGU, *trn*W-CCA, and *trn*Y-GUA.

### 3.6 Phylogenetic analysis

**Figure 6:**
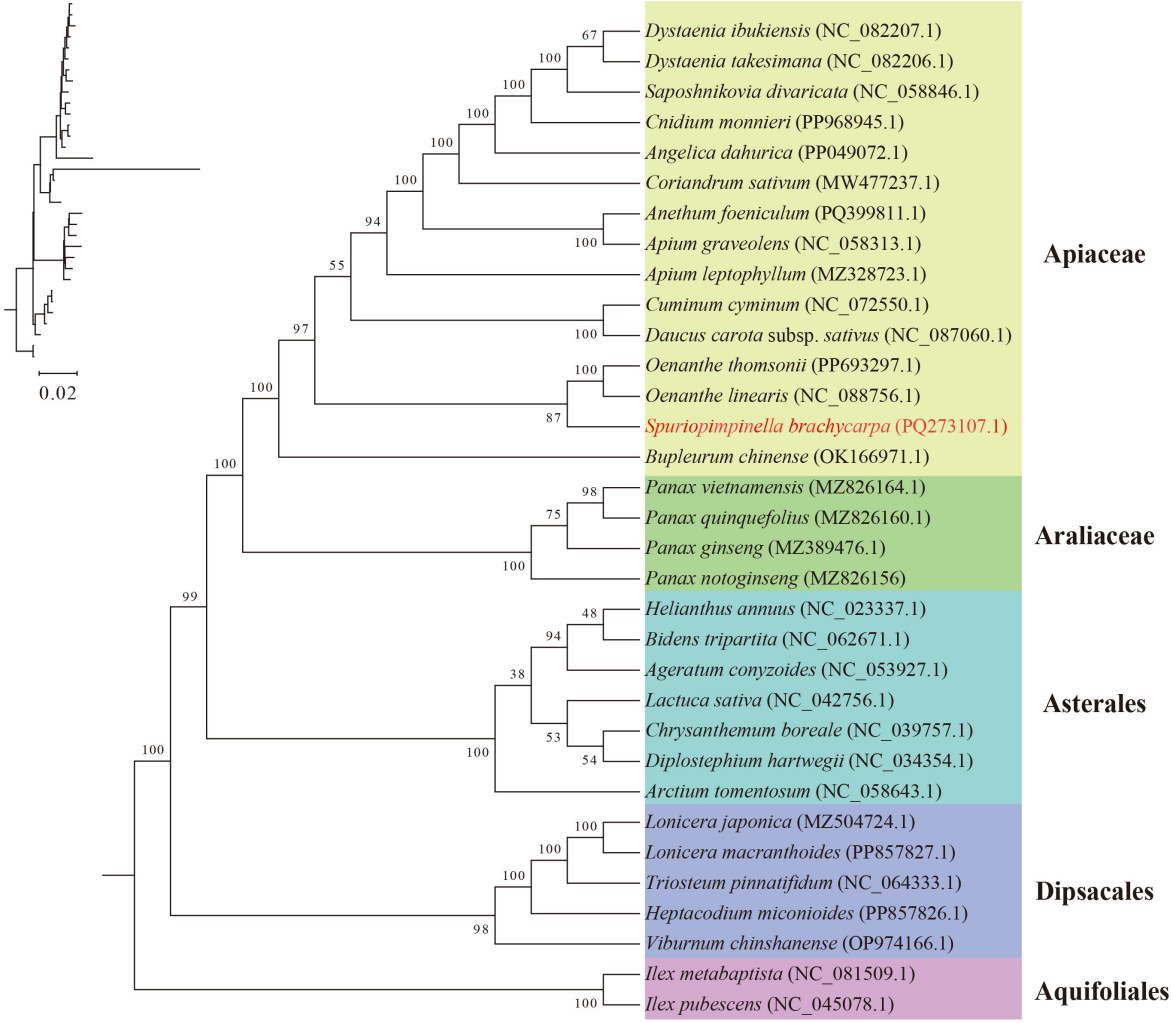
Phylogenetic relationships inferred from 33 mitochondrial protein-coding gene sequences.

Mitogenome data for species closely related to *S. brachycarpa* is limited. Within the Apiales order, 19 mitogenomes had been completely sequenced and deposited in NCBI, including 15 species from the Apiaceae family and four from the Araliaceae family. To better understand the phylogenetic relationships of *S. brachycarpa*, we expanded the analysis by incorporating mitogenome sequences from 33 species across the Apiales, Asterales, Dipsacales, and Aquifoliales orders, in which included all previously published sequences from the Apiales. After removing ambiguously aligned regions, a total of 30 shared protein-coding genes were aligned, totaling 25,820 bp in length, comprising 734 parsimony-informative sites (**Supplementary Table S5**). The phylogenetic topology revealed four monophyletic lineages at the order level with strong support values (BS > 98), of which Aguifoliales was the outgrop (**Figure 6**). Within Apiales, the topology strongly supported the reciprocally monophyletic clade of the two family arrangement, namely Apiaceae and Araliaceae (BS = 100). Moreover, *S. brachycarpa* was closely related to the clade that comprised *O. linearis* and *O. thomsonii* from Apiaceae, with moderate support (BS = 87).

### 3.7 Collinearity analysis

**Figure 7:**
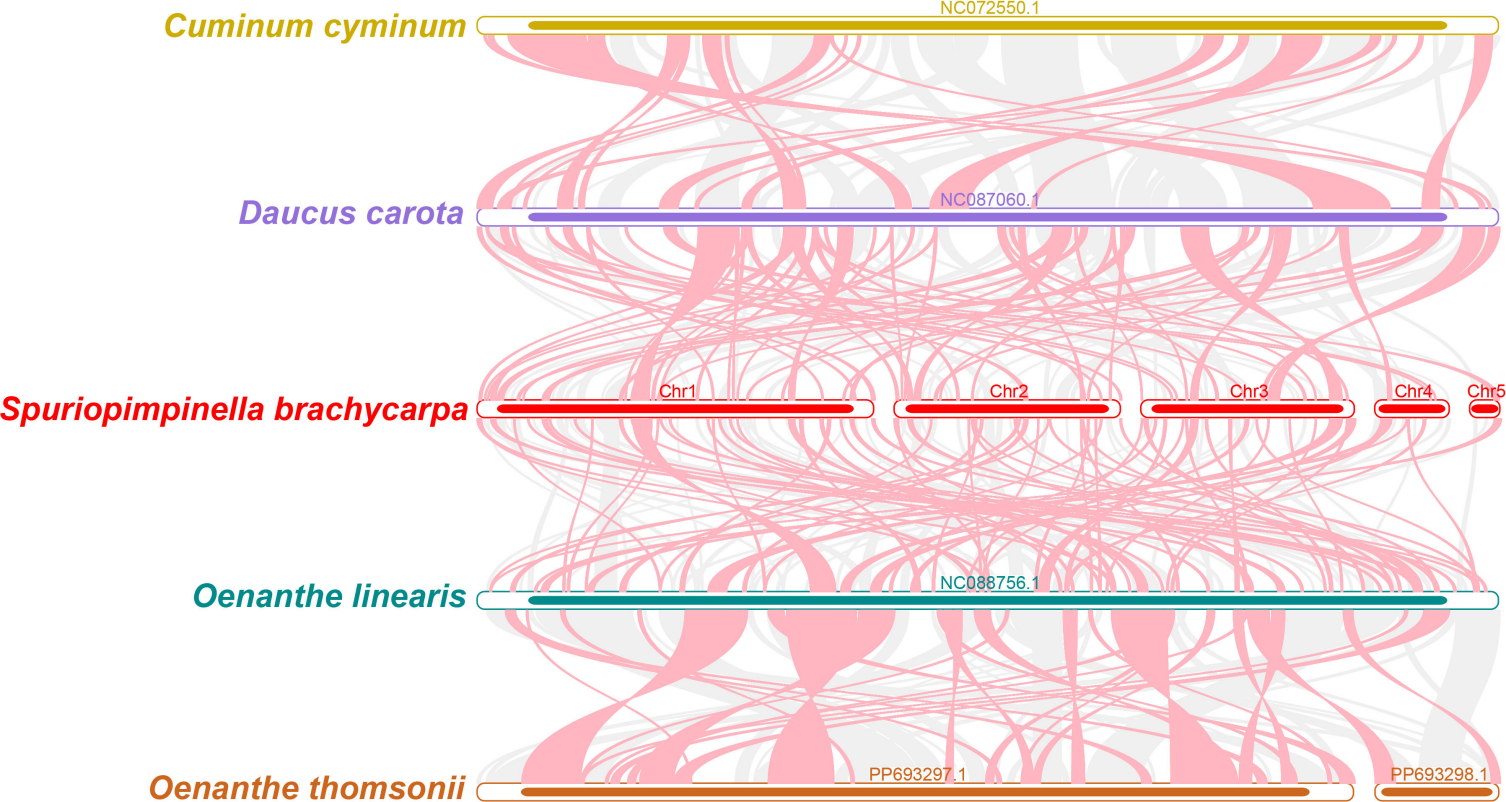
Collinearity analysis of *S. brachycarpa* and four closely related species. Red arcs highlight inverted regions, while gray arcs indicate homologous regions with high similarity.

Using the mitogenome of *S. brachycarpa* as a reference, we analyzed the collinear relationships among *C. cyminum*, *D. carota* subsp. *sativus*, *O. linearis*, and *O. thomsonii*. The analysis revealed numerous homologous collinear blocks, although these blocks were relatively short (**Figure 7**). Additionally, we identified distinct gaps representing regions that are unique to *S. brachycarpa*, with no homologous sequences found in the other species. The results showed inconsistent collinear block arrangements among the six mitogenomes, indicating that *S. brachycarpa* has undergone significant genomic rearrangements compared to its close relatives. Furthermore, the mitogenomes of the five Apiaceae species displayed a high degree of structural variability, suggesting that their sequence arrangements are extremely non-conservative.

## 4 Discussion

Plants are indispensable to human society, serving as vital sources of food, medicine, and economic value (Gray et al., 1999; Wallace, 1999; Poole and Penny, 2007). Understanding the genetic foundations that contribute to these valuable traits is crucial for maximizing their potential. Mitogenomes, in particular, play a significant role in revealing how plants adapt and evolve, given their involvement in essential cellular processes like energy production, respiration, and stress responses (Houten and Auwerx, 2004; Koonin, 2010), offering insights into the diversification and functional significance of various species (Gualberto et al., 2014; Gualberto and Newton, 2017). *S. brachycarpa* emerges as a species of considerable economic and medicinal value within the Apiaceae family. It is recognized as a valuable species within the *Spuriopimpinella* genus, which has been reclassified as an independent genus based on both molecular and morphological evidence (Downie et al., 2010; Wang et al., 2013). In this study, we sequenced, assembled, and reported, for the first time, the complete mitogenome of *S. brachycarpa*, providing novel insights into the unique properties of this valuable plant.

Plant mitogenomes are known to vary greatly in size, ranging from as small as 6.6 kb in some Plasmodium species to nearly 19 Mb in *Cathaya argyrophylla* (Hikosaka et al., 2010; Huang et al., 2024b). Such variation is often attributed to differences in non-coding regions and the presence of repetitive sequences (Gualberto et al., 2014; Gualberto and Newton, 2017), and mitogenome size is often correlated with specific evolutionary adaptations and properties (Huang et al., 2024b). In this study, the mitogenome of *S. brachycarpa* was assembled to a total length of 523,512 bp. This size is notably larger than those of closely related species such as *C. cyminum* (246,721 bp), *D. carota* subsp. *sativus* (250,368 bp), *O. linearis* (367,287 bp), and *O. thomsonii* (384,782 bp). This difference suggests unique evolutionary and functional characteristics in *S. brachycarpa*. To explore the reasons behind the larger mitogenome of *S. brachycarpa*, we compared coding sequence lengths, non-coding regions, and the abundance of LSRs among five closely related species. The total coding sequence lengths were 35,958 bp (15.57%), 37,321 bp (14.91%), 38,693 bp (10.57%), 43,410 bp (11.28%), and 43,492 bp (8.31%) in *C. cyminum*, *D. carota* subsp. *sativus*, *O. linearis*, *O. thomsonii*, and *S. brachycarpa*, respectively. We identified 209 LSRs totaling 12,039 bp in *C. cyminum*, 303 LSRs (15,223 bp) in *D. carota* subsp. *sativus*, 492 LSRs (33,095 bp) in *O. linearis*, 599 LSRs (57,329 bp) in *O. thomsonii*, and 3,155 LSRs (213,335 bp) in *S. brachycarpa*. Among the 3,155 LSRs in *S. brachycarpa*, 3,144 (99.65%) were located in non-coding regions. While the number of genes and the lengths of coding sequences were relatively consistent across species, *S. brachycarpa* exhibited a significant expansion in non-coding regions due to an abundance of LSRs. Repetitive sequences, such as inverted, palindromic, and direct repeats, are known to influence genome size, gene arrangement, and evolutionary dynamics in plant mitogenomes, as well as contribute to cytoplasmic male sterility and impact pollen development, which is vital for seedling cultivation and genetic improvement (Smith and Keeling, 2015; Dong et al., 2018; Martins et al., 2019; Yang et al., 2023; Wang et al., 2024). The extensive presence of LSRs in *S. brachycarpa* suggests that they have played a significant role in the expansion and structural complexity of its mitogenome. This observation indicates that LSR expansion may be a key driver of mitogenome size variation in *S. brachycarpa*.

RNA editing can affect gene expression and protein function, potentially influencing mitochondrial activity and plant adaptation (Gray et al., 1999; Mackenzie and McIntosh, 1999). The number of RNA editing sites in land plant mitogenomes varies widely, from none in *Marchantia polymorpha* to 2152 in *Selaginella moellendorffii* (Zhang et al., 2020; Lai et al., 2022). In *S. brachycarpa*, 618 C-to-U editing sites were detected across 30 protein-coding genes, demonstrating significant gene-specific variability. Most of these sites were located at the first and second codon positions, with 95.63% leading to amino acid changes. These edits, especially those in energy-related genes, may enhance mitochondrial efficiency and contribute to the plant’s distinct traits. The conserved C-to-U conversion pattern across Apiaceae suggests a shared RNA editing mechanism within the family, potentially reflecting an adaptive advantage. Frequent edits at the second codon position could play a key role in modulating protein structure, hydrophobicity, and stability (Jiang et al., 2022; Wu et al., 2022). Similarly, codon usage also plays an essential role in shaping mitogenome evolution and adaptation. In *S. brachycarpa* and related species, codon usage is influenced by factors such as gene expression level, gene length, tRNA abundance, and codon position, which further drive species-specific adaptation (Li et al., 2023). The shared preference for the codon GCU in *S. brachycarpa*, *O. linearis*, and *O. thomsonii* suggests conserved translational selection pressures within these closely related species of the Apiaceae family. Conversely, *C. cyminum* and *D. carota* subsp. *sativus* displayed the highest preference for the stop codon UAA, consistent with previous findings (Chen et al., 2024). In the mitogenome of *S. brachycarpa*, 28 codons were identified as high frequency, with 27 ending in A/T; while the ratios for *C. cyminum*, *D. carota* subsp. *sativus*, *O. linearis*, and *O. thomsonii* were 28/30, 28/30, 27/29, and 28/30, respectively. The predominance of codons ending in either A or T, with RSCU values of 1.00 or higher, highlights a strong AT bias at the third codon position, a common pattern in higher plants (Yang et al., 2021).

The evolutionary patterns of plant mitogenomes differ from those of animals, with lower mutation rates and frequent integration of foreign DNA, including plastome sequences (Zhang, 1995). This gene transfer is crucial for biological evolution, adaptation, and diversity (Xiong et al., 2008). In *S. brachycarpa*, we identified 59 homologous fragments between the mitochondrial and chloroplast genomes, totaling 42,553 bp and representing 8.13% of the mitogenome. These findings suggest horizontal gene transfer events, which are common in plant mitogenomes and contribute to genomic diversity and functional adaptation (Smith and Keeling, 2015; Kozik et al., 2019). Such transfers could influence the functional repertoire of the mitogenome and impact metabolic pathways relevant to the plant’s economic and medicinal value.

Further, our phylogenetic analysis, based on shared mitochondrial protein-coding genes, confirmed the placement of *S. brachycarpa* within the Apiaceae family, revealing its close relationships. This finding not only supports the taxonomic position of *S. brachycarpa* but also aligns with previous molecular studies on Apiaceae phylogeny (Downie et al., 2010; Wang et al., 2013). Previous phylogenetic analyses inferred from cpDNA sequences indicated that *Pimpinella brachycarpa* should be placed in the genus *Spuriopimpinella* (Wang et al., 2013), which is consistent with our result. Given the limited mitogenomic reports for Apiaceae, we also reconstructed a phylogenetic tree using *rps*16 and *rpl*16 intron sequences from the newly sequenced plastome, along with previously available data from Wang et al. (2013) (**Supplementary Figure S3**). The results resolved that two accessions of *S. brachycarpa* formed a clade and is sister to *S. arguta*, with a strong support value (BS = 100). Here, as the shared mitochondrial genes generated a higher-resolution topology compared with short DNA fragment, a more comprehensive phylogenetic tree can be further constructed based on extending sampling with mitogenome data.

Mitogenomes play a crucial role in energy metabolism, stress responses, and other essential cellular processes (Houten and Auwerx, 2004; Koonin, 2010). Alterations in mitogenome structure and function can have significant effects on plant physiology and adaptation, potentially influencing traits that are valuable to humans. Our findings provide a foundational understanding of the mitogenome of *S. brachycarpa*, highlighting its unique features and evolutionary trajectory. The complex genomic structure, characterized by extensive gene rearrangements, an abundance of LSRs leading to an enlarged genome size, and evidence of horizontal gene transfer through MTPTs, suggests a dynamic unique evolutionary history. The high number of RNA editing sites further underscores the potential for functional adaptations within the mitogenome. These findings suggest that the unique characteristics of the *S. brachycarpa* mitogenome may be linked to its economic and medicinal value. Further functional studies are necessary to elucidate the specific roles of these mitogenome features in *S. brachycarpa* and their contributions to its valuable traits. Understanding these connections could provide valuable insights into the genetic basis of the plant’s medicinal properties and inform breeding and conservation strategies. In conclusion, our comprehensive analysis of the mitogenome of *S. brachycarpa* provides valuable insights into its evolutionary processes, genetic diversity, and potential links to its unique economic and medicinal properties. This research not only enhances our understanding of mitogenome evolution in the Apiaceae family but also underscores the significance in the study of plant biology and its applications in agriculture and medicine.

## 5 Conclusion

This study presents a comprehensive analysis of the mitogenome of *S. brachycarpa*. Leveraging both second and third-generation high-throughput sequencing technologies, we successfully assembled and annotated the complex multi-chromosome mitogenome, which spans 523,512 bp with a GC content of 43.37%. The genome includes 30 unique protein-coding genes, 21 tRNA genes, and three rRNA genes. Comparative analysis revealed that *S. brachycarpa* shares some collinear features with other Apiaceae species. However, it also exhibits significant gene location rearrangements and structural variations. These findings suggest that *S. brachycarpa* possesses one of the most complex genome structures among the analyzed Apiaceae species, characterized by relatively short homologous regions. Additionally, we identified 618 potential RNA editing sites, all of which involved C-to-U editing. Fifty-nine homologous fragments between the mitogenome and plastome were discovered spanning 42,553 bp and constituting 8.13% of the mitogenome. Overall, this study underscores the uniqueness and complexity of the *S. brachycarpa* mitogenome, offering valuable insights into the evolutionary relationships and genetic diversity within the Apiaceae family. These findings not only enhance the existing genomic database for Apiaceae but also provide a solid theoretical foundation for future research in molecular systematics and conservation genetics.

## Data Availability

The complete sequences of the mitogenome and plastome of *S. brachycarpa* are available in the GenBank nucleotide database. The accession number for the plastome is PQ213365, while the accession numbers for the mitogenome chromosomes are filed under accession numbers PQ273107 to PQ273111. Additionally, the sequencing reads used in the assembly for this study are deposited in the NCBI repository under the following identifiers: BioProject PRJNA1149306, BioSample SAMN43240799, and Sequence Read Archive (SRA) data SRR30284393 to SRR30284395.

## Supplementary material

Supplementary Figure 1 The morphological characteristics of *S. brachycarpa.*

Supplementary Figure 2 The plastome map of *S. brachycarpa.*

Supplementary Figure 3 Phylogenetic relationships inferred from *rpl*16 and *rps*16 intron sequences.

Supplementary Table 1 Analysis of repeat regions supporting multi-copy genes in ONT Data.

Supplementary Table 2 Genes predicted in the plastome of *S. brachycarpa.*

Supplementary Table 3 RNA editing sites identified in *S. brachycarpa* and four related species.

Supplementary Table 4 RSCU values in *S. brachycarpa* and four related species.

Supplementary Table 5 Number of conserved genes among 33 species.

## References

[B1] Arrieta-Montiel M. P. Mackenzie S. A. (2011). “Plant Mitochondrial Genomes and Recombination,” in Plant Mitochondria, vol. 1 . Ed. Kempken F. (Springer, New York, NY, USA), 65–82.

[B2] Benson G. (1999). Tandem repeats finder: A program to analyze DNA sequences. Nucleic Acids Res. 27, 573–580. doi: 10.1093/nar/27.2.573 9862982 PMC148217

[B3] Boissieu H. (1906). Note sur quelques Ombellifères de la Chine, d’après les collections du Muséum d’Histoire naturelle de Paris. Bull. Soc Bot. Fr. 53, 428. doi: 10.1080/00378941.1906.10831189

[B4] Chen L. Dong X. Huang H. Xu H. Rono P. C. Cai X.. (2024). Assembly and comparative analysis of the initial complete mitochondrial genome of *Primulina hunanensis* (Gesneriaceae): a cave-dwelling endangered plant. BMC Genomics 25, 322. doi: 10.1186/s12864-024-10247-9 38561677 PMC10983754

[B5] Chen Z. W. Zhao N. Li S. S. Grover C. E. Nie H. S. Wendel J. F.. (2017). Plant mitochondrial genome evolution and cytoplasmic male sterility. Crit. Rev. Plant Sci. 36, 55–69. doi: 10.1080/07352689.2017.1327762

[B6] Clarkson J. J. Zuntini A. R. Maurin O. Downie S. R. Plunkett G. M. Nicolas A. N.. (2021). A higher-level nuclear phylogenomic study of the carrot family (Apiaceae). Am. J. Bot. 108, 1252–1269. doi: 10.1002/ajb2.1701 34287829

[B7] Danecek P. McCarthy S. A. (2017). BCFtools/csq: haplotype-aware variant consequences. Bioinformatics 33, 2037–2039. doi: 10.1093/bioinformatics/btx100 28205675 PMC5870570

[B8] Dong S. Zhao C. Chen F. Liu Y. Zhang S. Wu H.. (2018). The complete mitochondrial genome of the early flowering plant *Nymphaea colorata* is highly repetitive with low recombination. BMC Genomics 19, 614. doi: 10.1186/s12864-018-4957-6 30107780 PMC6092842

[B9] Downie S. R. Spalik K. Katz-Downie D. S. Reduron J.-P. (2010). Major clades within Apiaceae subfamily Apioideae as inferred by phylogenetic analysis of nrDNA ITS sequences. Plant Divers. Evol. 128, 111–136. doi: 10.1127/1869-6155/2010/0128-0005

[B10] Dunn N. A. Unni D. R. Diesh C. Munoz-Torres M. Harris N. L. Yao E.. (2019). Apollo: democratizing genome annotation. PloS Comput. Biol. 15, e1006790. doi: 10.1371/journal.pcbi.1006790 30726205 PMC6380598

[B11] Dyall S. D. Brown M. T. Johnson P. J. (2004). Ancient invasions: from endosymbionts to organelles. Science 304, 253–257. doi: 10.1126/science.1094884 15073369

[B12] Edera A. A. Small I. Milone D. H. Sanchez-Puerta M. V. (2021). Deepred-Mt: Deep representation learning for predicting C-to-U RNA editing in plant mitochondria. Comput. Biol. Med. 136, 104682. doi: 10.1016/j.compbiomed.2021.104682 34343887

[B13] Govaerts R. Nic Lughadha E. Black N. Turner R. Paton A. (2021). The World Checklist of Vascular Plants, a continuously updated resource for exploring global plant diversity. Sci. Data 8, 215. doi: 10.1038/s41597-021-00997-6 34389730 PMC8363670

[B14] Gray M. W. Burger G. Lang B. F. (1999). Mitochondrial evolution. Science 283, 1476–1481. doi: 10.1126/science.283.5407.1476 10066161

[B15] Gualberto J. M. Mileshina D. Wallet C. Niazi A. K. Weber-Lotfi F. Dietrich A. (2014). The plant mitochondrial genome: Dynamics and maintenance. Biochimie 100, 107–120. doi: 10.1016/j.biochi.2013.09.016 24075874

[B16] Gualberto J. M. Newton K. J. (2017). Plant mitochondrial genomes: dynamics and mechanisms of mutation. Annu. Rev. Plant Biol. 68, 225–252. doi: 10.1146/annurev-arplant-043015-112232 28226235

[B17] Hikosaka K. Watanabe Y. Tsuji N. Kita K. Kishine H. Arisue N. (2010). Divergence of the mitochondrial genome structure in the apicomplexan parasites, Babesia and Theileria, Mol. Biol. Evol. 27, 1107–1116. doi: 10.1093/molbev/msp320 20034997

[B18] Houten S. M. Auwerx J. (2004). PGC-1α: turbocharging mitochondria. J. Clin. Invest. 119, 5–7. doi: 10.1016/j.cell.2004.09.016 15454076

[B19] Huang K. Xu W. Hu H. Jiang X. Sun L. Zhao W.. (2024b). The mitochondrial genome of *Cathaya argyrophylla* reaches 18.99 Mb: Analysis of super-large mitochondrial genomes in Pinaceae. arXiv. Available at: https://arxiv.org/abs/2410.07006v1. 10.3389/fpls.2025.1556332 PMC12222119 40612618

[B20] Huang L. Yu H. Wang Z. Xu W. (2024a). CPStools: A package for analyzing chloroplast genome sequences. iMetaOmics, e25. doi: 10.1002/imo2.25 PMC12806284 41676121

[B21] Jiang H. Lu Q. Qiu S. Yu H. Wang Z. Yu Z.. (2022). Fujian cytoplasmic male sterility and the fertility restorer gene OsRf19 provide a promising breeding system for hybrid rice. Proc. Natl. Acad. Sci. U.S.A. 119, e2208759119. doi: 10.1073/pnas.2208759119 35969741 PMC9407659

[B22] Jiang M. Ni Y. Li J. Liu C. (2023). Characterisation of the complete mitochondrial genome of *taraxacum mongolicum* revealed five repeat-mediated recombinations. Plant Cell Rep. 42, 775–789. doi: 10.1007/s00299-023-02994-y 36774424

[B23] Jin J.-J. Yu W.-B. Yang J.-B. Song Y. dePamphilis C. W. Yi T.-S.. (2020). GetOrganelle: A fast and versatile toolkit for accurate *de novo* assembly of organelle genomes. Genome Biol. 21, 241. doi: 10.1186/s13059-020-02154-5 32912315 PMC7488116

[B24] Johnson M. Zaretskaya I. Raytselis Y. Merezhuk Y. McGinnis S. Madden T. L. (2008). NCBI BLAST: A better web interface. Nucleic Acids Res. 36, W5–W9. doi: 10.1093/nar/gkn201 18440982 PMC2447716

[B25] Karin B. R. Arellano S. Wang L. Walzer K. Pomerantz A. Vasquez J. M.. (2023). Highly-multiplexed and efficient long-amplicon PacBio and Nanopore sequencing of hundreds of full mitochondrial genomes. BMC Genomics. 24, 229. doi: 10.1186/s12864-023-09277-6 37131128 PMC10155392

[B26] Katoh K. Standley D. M. (2013). MAFFT multiple sequence alignment software version 7: improvements in performance and usability. Mol. Biol. Evol. 30, 772–780. doi: 10.1093/molbev/mst010 23329690 PMC3603318

[B27] Kitagawa M. (1941). Miscellaneous Notes on Apiaceae (Umbelliferae) of Japan and Manchuria (IV). J. Jpn. Bot. 17, 558. doi: 10.51033/jjapbot.17_10_2588

[B28] Kolmogorov M. Yuan J. Lin Y. Pevzner P. A. (2019). Assembly of long, error-prone reads using repeat graphs. Nat. Biotechnol. 37, 540–546. doi: 10.1038/s41587-019-0072-8 30936562

[B29] Koonin E. V. (2010). The origin and early evolution of eukaryotes in the light of phylogenomics. Genome Biol. 11, 209. doi: 10.1186/gb-2010-11-5-209 20441612 PMC2898073

[B30] Kozik A. Rowan B. A. Lavelle D. Berke L. Schranz M. E. Michelmore R. W.. (2019). The alternative reality of plant mitochondrial DNA: one ring does not rule them all. PloS Genet. 15, e1008373. doi: 10.1371/journal.pgen.1008373 31469821 PMC6742443

[B31] Krzywinski M. Schein J. Birol I. Connors J. Gascoyne R. Horsman D.. (2009). Circos: an information aesthetic for comparative genomics. Genome Res. 19, 1639–1645. doi: 10.1101/gr.092759.109 19541911 PMC2752132

[B32] Kurtz S. Choudhuri J. V. Ohlebusch E. Schleiermacher C. Stoye J. Giegerich R. (2001). REPuter: the manifold applications of repeat analysis on a genomic scale. Nucleic Acids Res. 29, 4633–4642. doi: 10.1093/nar/29.22.4633 11713313 PMC92531

[B33] Lai C. Wang J. Kan S. Zhang S. Li P. Reeve W. G.. (2022). Comparative analysis of mitochondrial genomes of *Broussonetia* spp. (Moraceae) reveals heterogeneity in structure, synteny, intercellular gene transfer, and RNA editing. Front. Plant Sci. 13, 1052151. doi: 10.3389/fpls.2022.1052151 36531410 PMC9751378

[B34] Li H. Durbin R. (2009). Fast and accurate short read alignment with burrows-wheeler transform. Bioinformatics 25, 1754–1760. doi: 10.1093/bioinformatics/btp324 19451168 PMC2705234

[B35] Li Y. Hu X. Xiao M. Huang J. Lou Y. Hu F.. (2023). An analysis of codon utilization patterns in the chloroplast genomes of three species of *coffea* . BMC Genomic Data 24, 42. doi: 10.1186/s12863-023-01108-w 37558997 PMC10413492

[B36] Li Y.-Y. Liu Y.-Y. Zeng X. Wu P. Li Q.-M. Guo S.-X.. (2024). Complete mitochondrial genome of *angelica dahurica* and its implications on evolutionary analysis of complex mitochondrial genome architecture in apiaceae. Front. Plant Sci. 15. doi: 10.3389/fpls.2024.1367299 PMC11074370 38716337

[B37] Lowe T. M. Eddy S. R. (1997). tRNAscan-SE: A program for improved detection of transfer RNA genes in genomic sequence. Nucleic Acids Res. 25, 955–964. doi: 10.1093/nar/25.5.955 9023104 PMC146525

[B38] Mackenzie S. McIntosh L. (1999). Higher plant mitochondria. Plant Cell 11, 571–586. doi: 10.1105/tpc.11.4.571 10213779 PMC144202

[B39] Mannella C. A. (2006). The relevance of mitochondrial membrane topology to mitochondrial function. Biochim. Biophys. Acta 1762, 140–147. doi: 10.1016/j.bbadis.2005.07.001 16054341

[B40] Margulis L. (1970). Origin of eukaryotic cells (New Haven: Yale University Press).

[B41] Martins G. Balbino E. Marques A. Almeida C. (2019). Complete mitochondrial genomes of the *Spondias tuberosa* Arr. Cam and *Spondias mombin* L. reveal highly repetitive DNA sequences. Gene 720, 144026. doi: 10.1016/j.gene.2019.144026 31377315

[B42] Nicholls D. G. Budd S. L. (2000). Mitochondria and neuronal survival. Physiol. Rev. 80, 315–360. doi: 10.1152/physrev.2000.80.1.315 10617771

[B43] Ou T. Wu Z. Tian C. Yang Y. Li Z. (2024). Complete mitochondrial genome of *Agropyron cristatum* reveals gene transfer and RNA editing events. BMC Plant Biol. 24, 830. doi: 10.1186/s12870-024-05558-8 39232676 PMC11373303

[B44] Pimenov M. G. Leonov M. V. (1993). The Genera of the Umbelliferae: A Nomenclator (Kew, UK: Royal Botanic Gardens).

[B45] Plunkett G. M. Pimenov M. G. Reduron J.-P. Kljuykov E. V. van Wyk B.-E. Ostroumova T. A.. (2018). “Apiaceae,” in Flowering Plants. Eudicots. Eds. Kadereit J. W. Bittrich V. (Springer International Publishing, Cham, Switzerland), 9–206.

[B46] Poole A. M. Penny D. (2007). Evaluating hypotheses for the origin of eukaryotes. Bioessays 29, 74–84. doi: 10.1002/bies.20516 17187354

[B47] Raimundo J. Reis C. M. G. Ribeiro M. M. (2018). Rapid, simple and potentially universal method for DNA extraction from *Opuntia* spp. Fresh cladode tissues suitable for PCR amplification. Mol. Biol. Rep. 45, 1405–1412. doi: 10.1007/s11033-018-4303-8 30109548

[B48] Scorrano L. Ashiya M. Buttle K. Weiler S. Oakes S. A. Mannella C. A.. (2002). A Distinct Pathway Remodels Mitochondrial Cristae and Mobilizes Cytochrome c during Apoptosis. Dev. Cell 2, 55–67. doi: 10.1016/s1534-5807(01)00116-2 11782314

[B49] Sheh M.-L. Pu F.-D. Pan Z.-H. Watson M. F. Cannon J. F. M. Holmes-Smith. (2005). “Apiaceae,” in Editorial Committee, Flora of China, vol. 14. (Missouri Botanical Garden Press, St. Louis, MI, USA), 1–205.

[B50] Shi L. Chen H. Jiang M. Wang L. Wu X. Huang L.. (2019). CPGAVAS2, an integrated plastome sequence annotator and analyzer. Nucleic Acids Res. 47, W65–W73. doi: 10.1093/nar/gkz345 31066451 PMC6602467

[B51] Smith D. R. Keeling P. J. (2015). Mitochondrial and plastid genome architecture: Reoccurring themes, but significant differences at the extremes. Proc. Natl. Acad. Sci. U.S.A. 112, 10177–10184. doi: 10.1073/pnas.1422049112 25814499 PMC4547224

[B52] Stamatakis A. (2014). RAxML version 8: a tool for phylogenetic analysis and post-analysis of large phylogenies. Bioinformatics 30, 1312–1313. doi: 10.1093/bioinformatics/btu033 24451623 PMC3998144

[B53] Štorchová H. Krüger M. (2024). Methods for assembling complex mitochondrial genomes in land plants. J. Exp. Bot. 75, 5169–5174. doi: 10.1093/jxb/erae034 38302086

[B54] Wallace D. C. (1999). Mitochondrial diseases in man and mouse. Science 283, 1482–1488. doi: 10.1126/science.283.5407.1482 10066162

[B55] Wang Z. X. Downie S. R. Tan J. B. Liao C. Y. Yu Y. He X. J. (2013). Molecular phylogenetics of *Pimpinella* and allied genera (Apiaceae), with emphasis on Chinese native species, inferred from nrDNA ITS and cpDNA intron sequence data. Nord. J. Bot. 32, 642–657. doi: 10.1111/j.1756-1051.2013.00343.x

[B56] Wang J. Kan S. Liao X. Daniell H. Jin S. Wu Z. (2024). Plant organellar genomes: much done, much more to do. Trends Plant Sci. 29, 754–769. doi: 10.1016/j.tplants.2023.12.014 38220520

[B57] Wang Y. Tang H. Debarry J. D. Tan X. Li J. Wang X.. (2012). MCScanX: A toolkit for detection and evolutionary analysis of gene synteny and collinearity. Nucleic Acids Res. 40, e49. doi: 10.1093/nar/gkr1293 22217600 PMC3326336

[B58] Wick R. R. Judd L. M. Gorrie C. L. Holt K. E. (2017). Unicycler: resolving bacterial genome assemblies from short and long sequencing reads. PloS Comput. Biol. 13, e1005595. doi: 10.1371/journal.pcbi.1005595 28594827 PMC5481147

[B59] Wick R. R. Schultz M. B. Zobel J. Holt K. E. (2015). Bandage: interactive visualization of *de novo* genome assemblies. Bioinformatics 31, 3350–3352. doi: 10.1093/bioinformatics/btv383 26099265 PMC4595904

[B60] Wu J. J. Cao Z. Hassan S. S. U. Zhang H. Ishaq M. Yu X.. (2023). Emerging biopharmaceuticals from *pimpinella* genus. Molecules 28, 1571. doi: 10.3390/molecules28041571 36838559 PMC9959726

[B61] Wu Z. Q. Liao X. Z. Zhang X. N. Tembrock L. R. Broz A. (2022). Genomic architectural variation of plant mitochondria - a review of multichromosomal structuring. J. Syst. Evol. 60, 160–168. doi: 10.1111/jse.12655

[B62] Xiong A. S. Peng R. H. Zhuang J. Gao F. Zhu B. Fu X. Y.. (2008). Gene duplication and transfer events in plant mitochondria genome. Biochem. Biophys. Res. Commun. 376, 427–430. doi: 10.1016/j.bbrc.2008.08.116 18765231

[B63] Yang H. Li W. Yu X. Zhang X. Zhang Z. Liu Y.. (2021). Insights into molecular structure, genome evolution and phylogenetic implication through mitochondrial genome sequence of *Gleditsia sinensis* . Sci. Rep. 11, 14850. doi: 10.1038/s41598-021-93920-9 34290263 PMC8295344

[B64] Yang H. Ni Y. Zhang X. Li J. Chen H. Liu C. (2023). The mitochondrial genomes of *panax notoginseng* reveal recombination mediated by repeats associated with DNA replication. Int. J. Biol. Macromol. 252, 126359. doi: 10.1016/j.ijbiomac.2023.126359 37619687

[B65] Zhang S. H. (1995). The structural differences between animal and plant mitochondrial genomes - two evolutionary scenarios. Zool. Res. 16, 132–145.

[B66] Zhang J. Fu X. X. Li R. Q. Zhao X. Liu Y. Li M. H.. (2020). The hornwort genome and early land plant evolution. Nat. Plants 6, 107–118. doi: 10.1038/s41477-019-0588-4 32042158 PMC7027989

[B67] Zheng Y. (2016). Analysis of influencing factors of *Spuriopimpinella brachycarpa’s* growth and nutritional quality under canopy in Eastern-Liaoning. Shenyang Agricultural University, Shenyang, China.

