# Supplementary figures and images for: Structural and evolutionary analyses of the mitochondrial genome of Spuriopimpinella brachycarpa
Source: Front Plant Sci. 2024 Nov 26;15:1492723. doi: 10.3389/fpls.2024.1492723 (PMC11628310; doi:10.3389/fpls.2024.1492723)

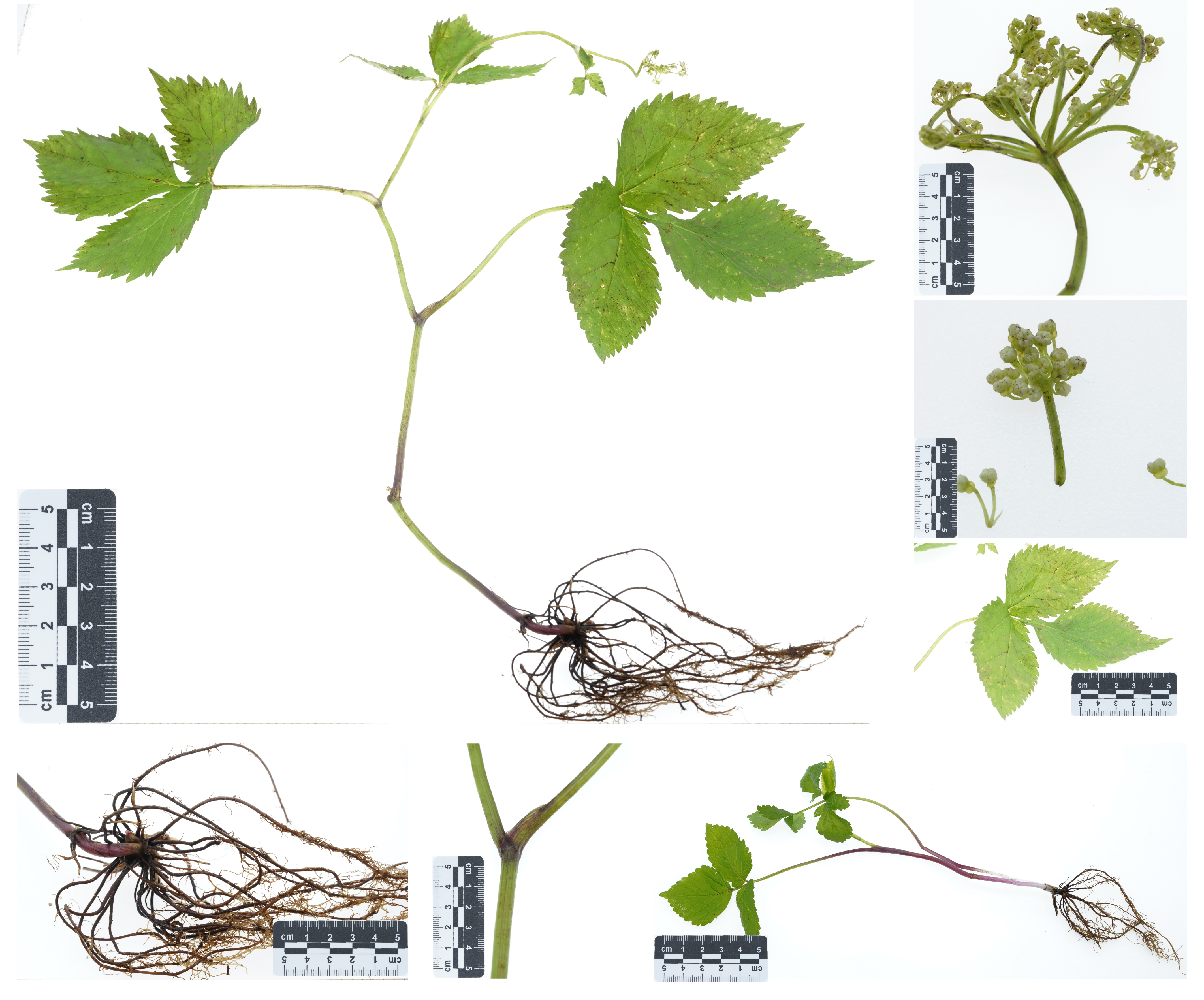

Supplement: Supplementary Figure 1 — The morphological characteristics of S. brachycarpa. [file Image1.jpeg]

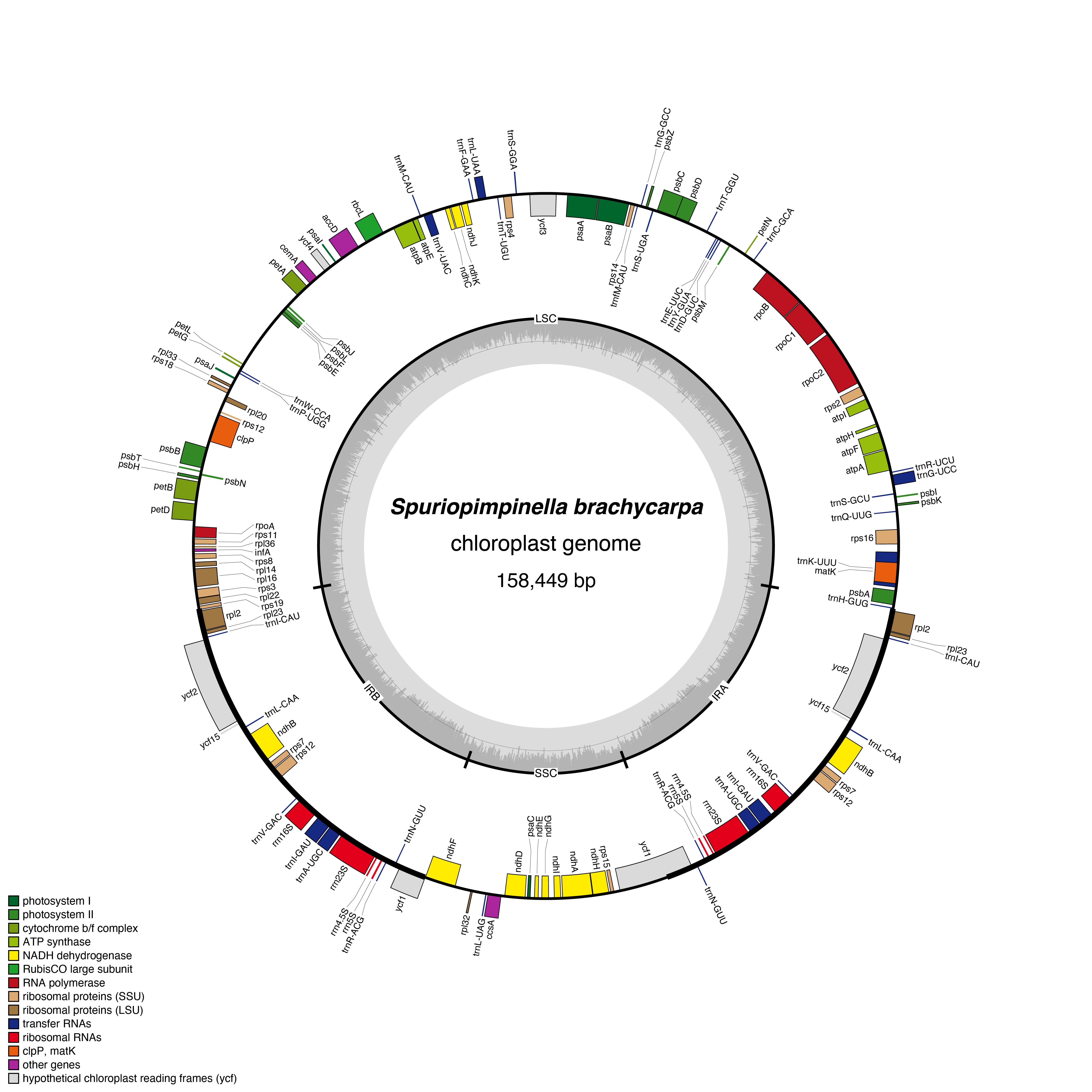

Supplement: Supplementary Figure 2 — The plastome map of S. brachycarpa. [file Image2.jpeg]

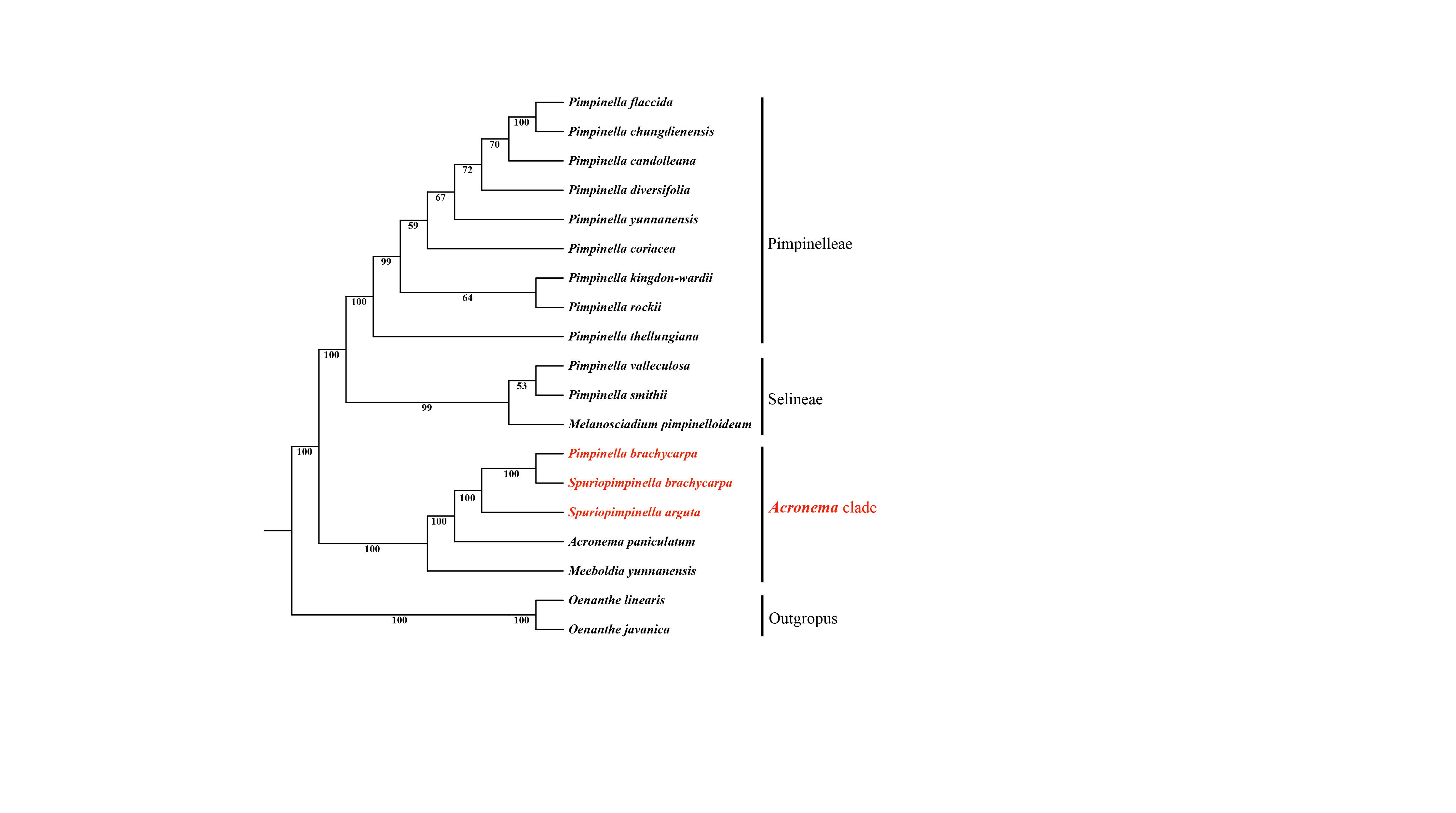

Supplement: Supplementary Figure 3 — Phylogenetic relationships inferred from rpl16 and rps16 intron sequences. [file Image3.jpeg]

Table S1 Analysis of repeat regions supporting multi-copy genes in ONT Data


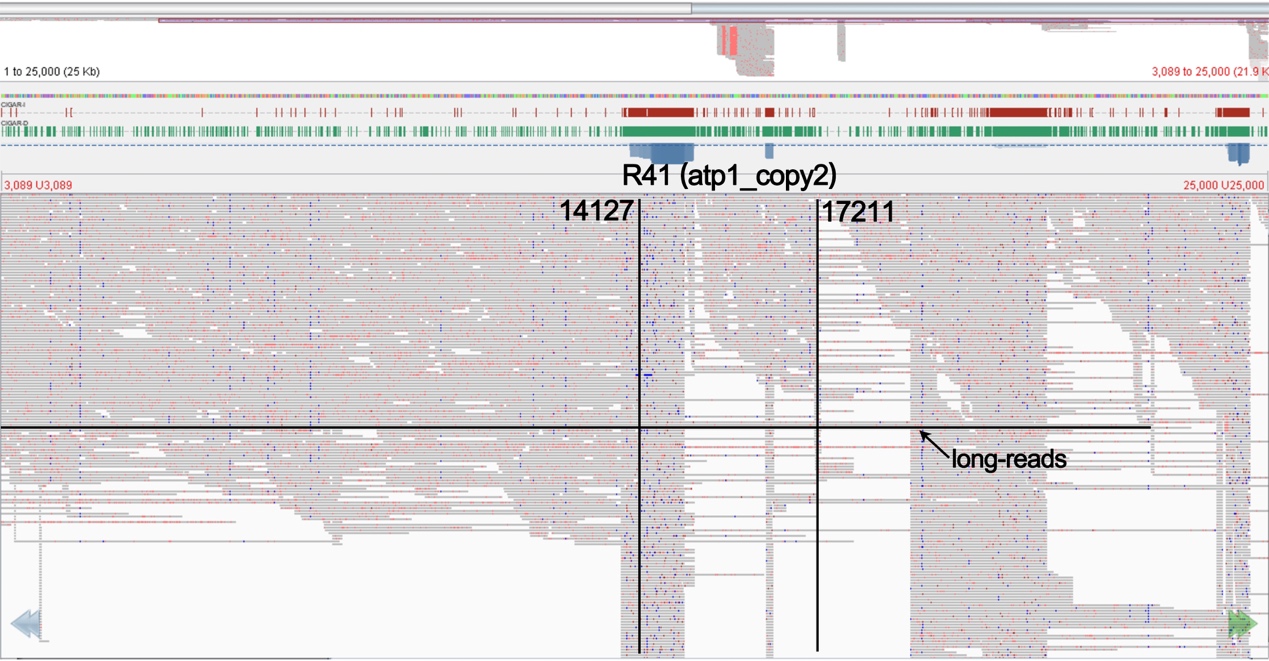


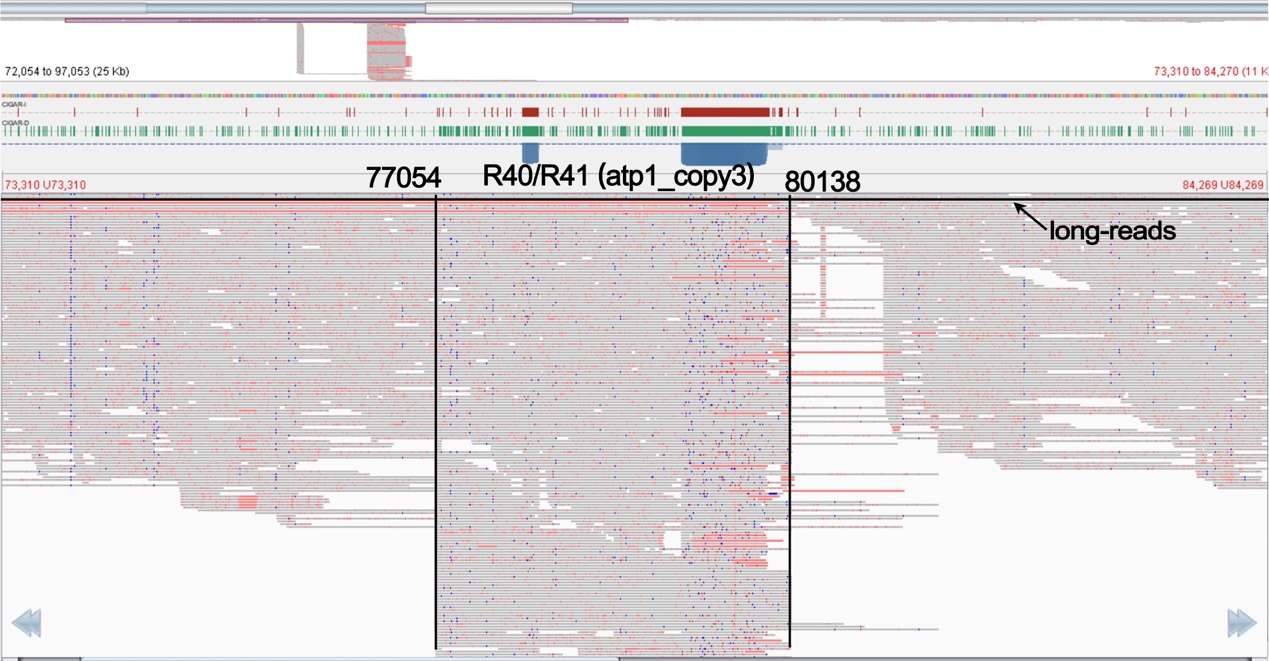

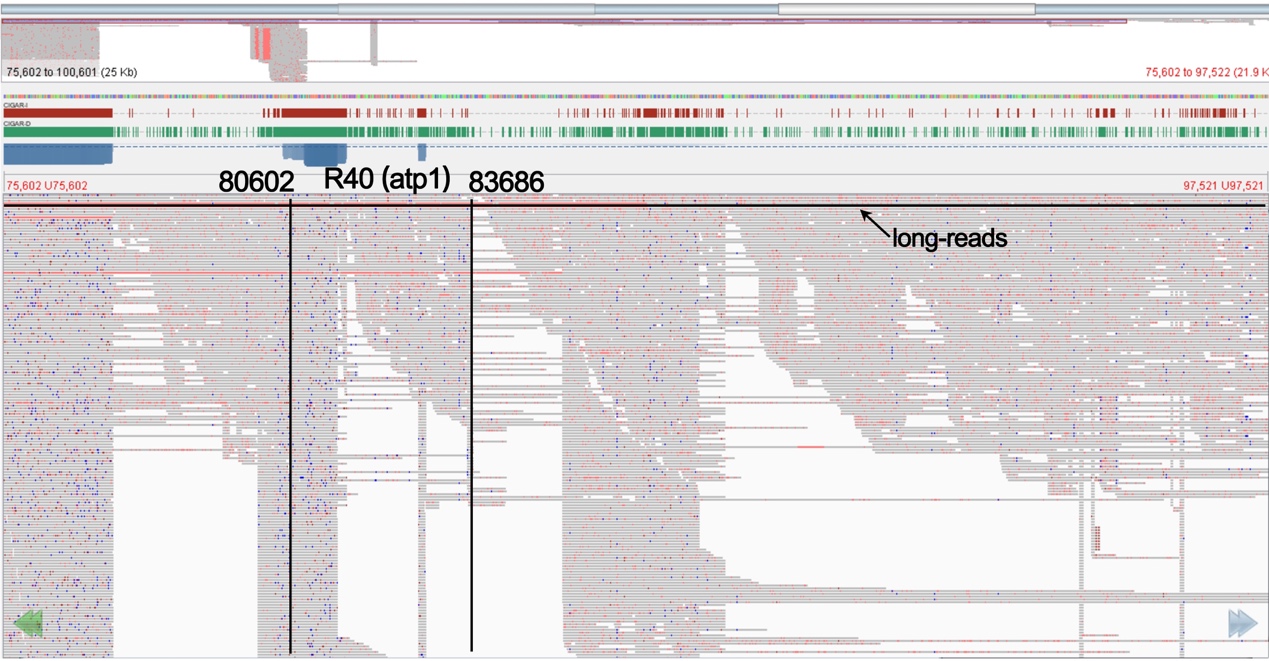

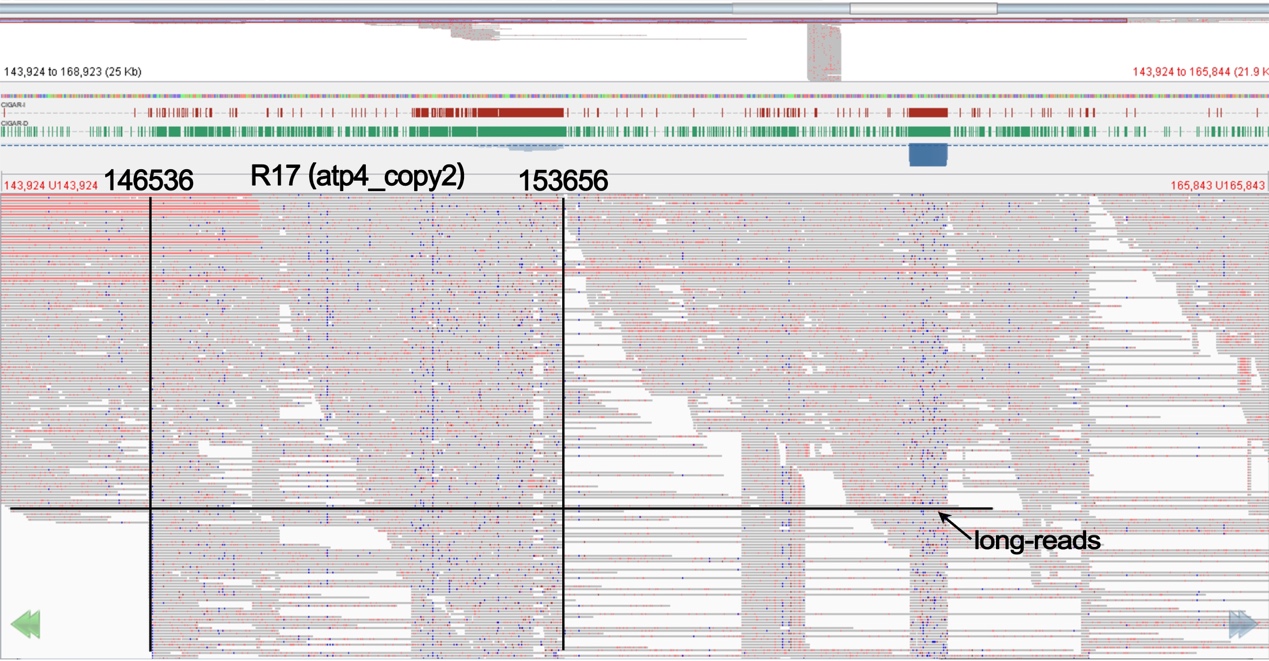

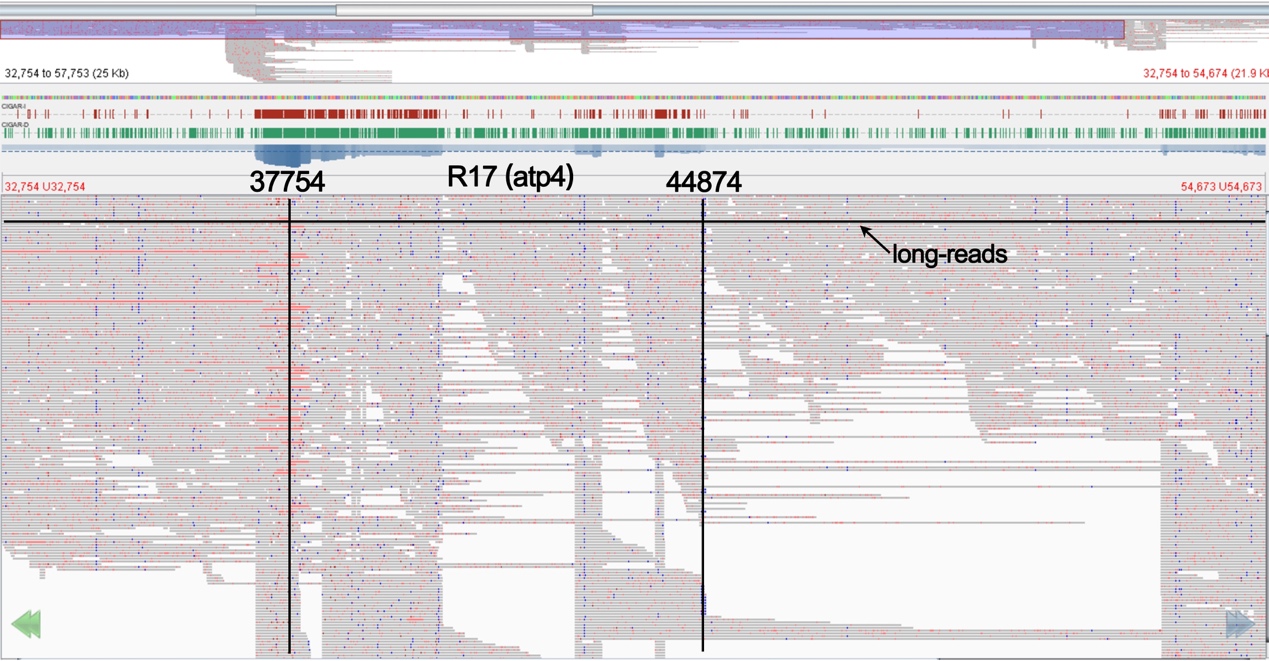

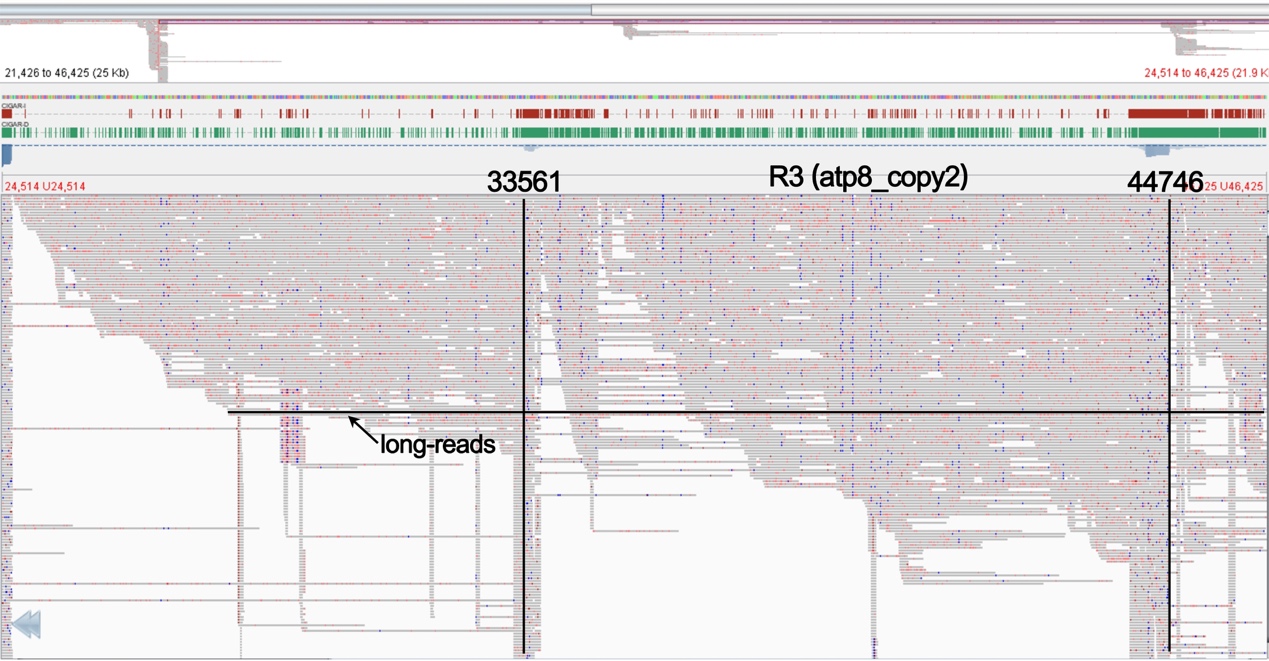

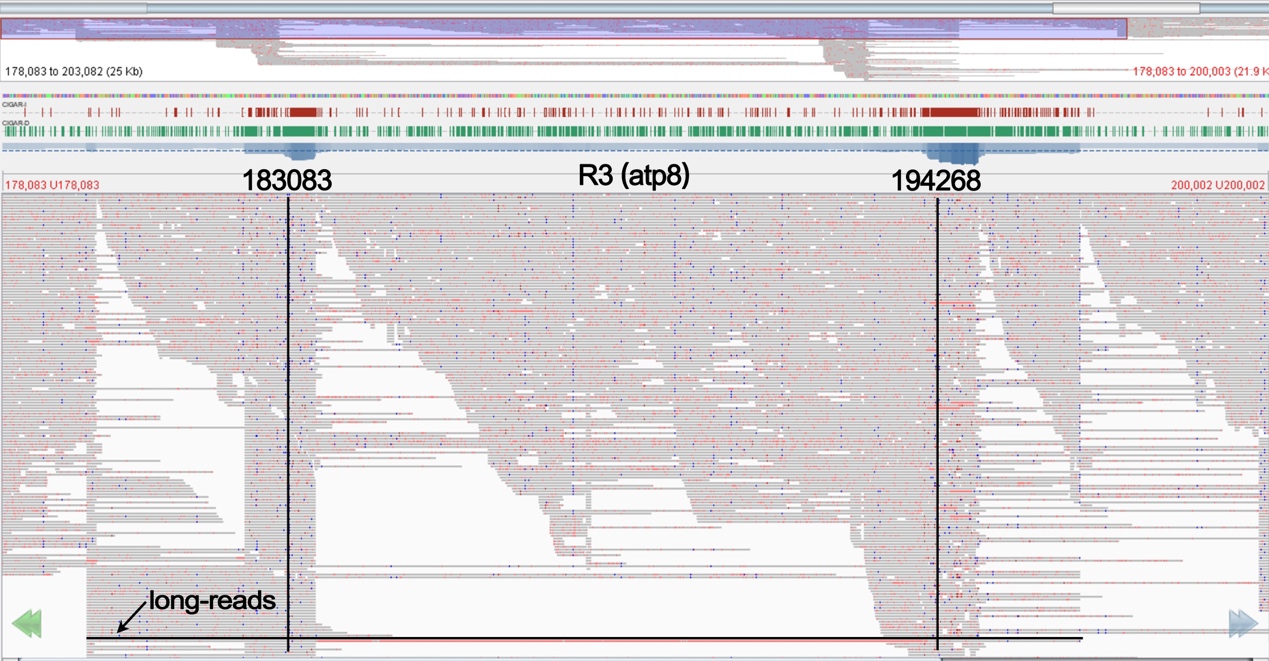

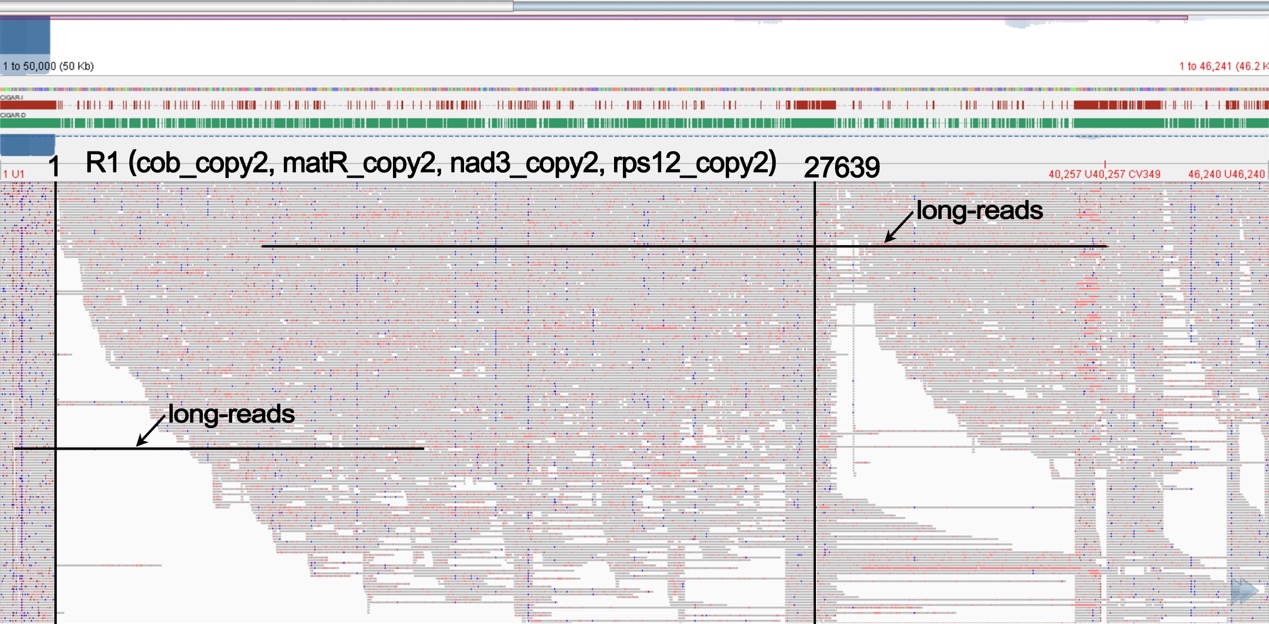

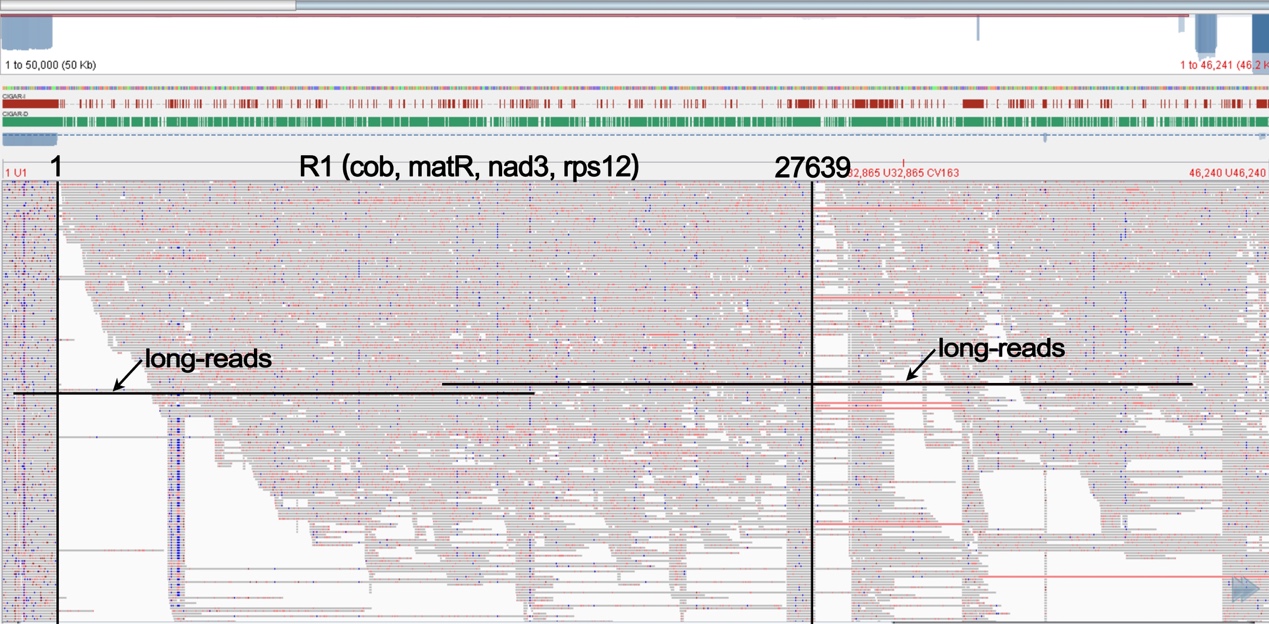

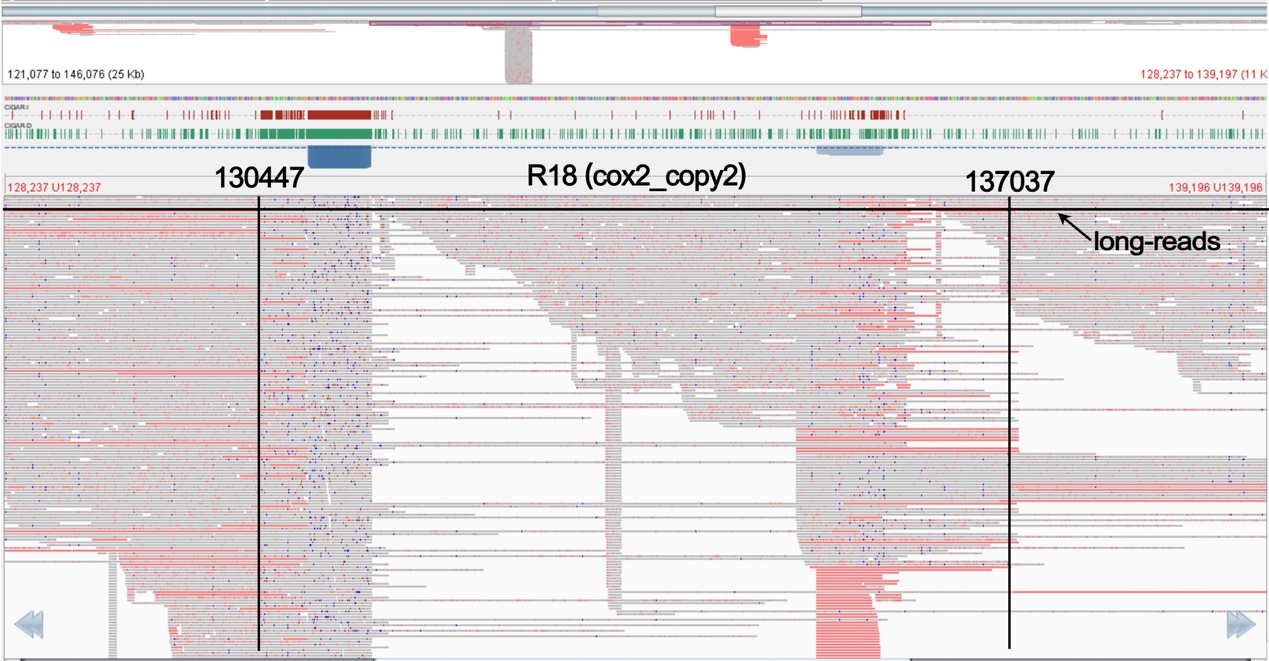

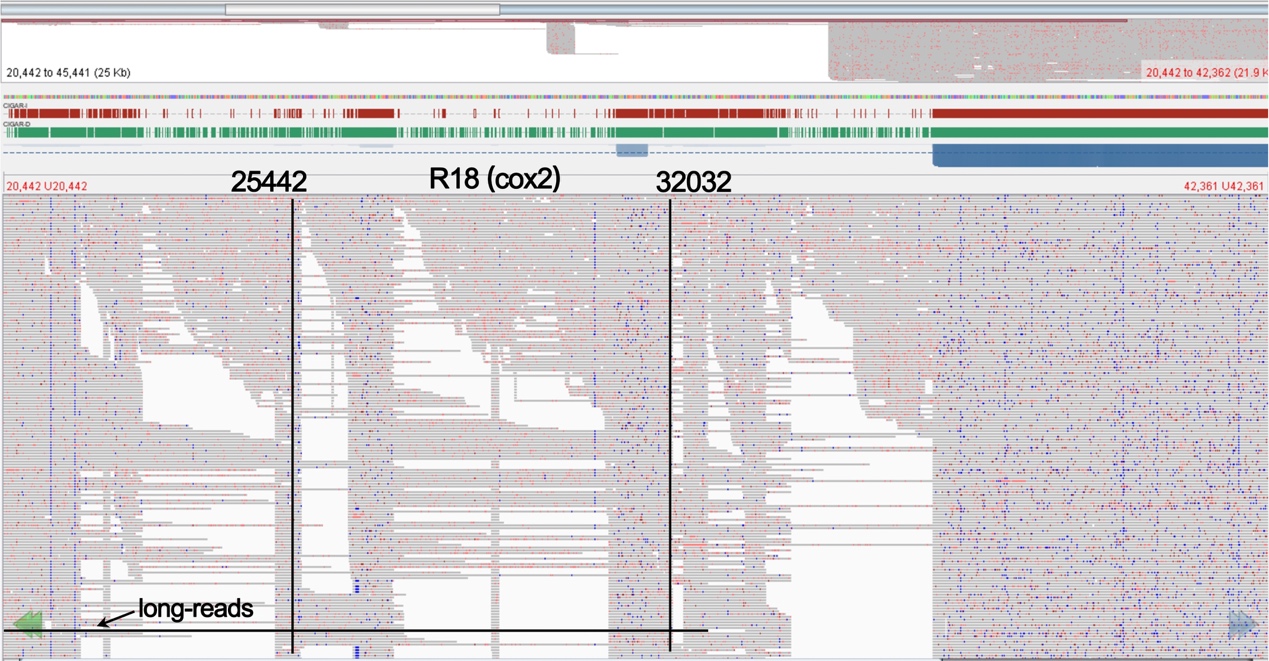

Supplement: Supplementary Table 1 — Analysis of repeat regions supporting multi-copy genes in ONT Data. [file Table1.docx]
