# Supplementary material for: Structural and evolutionary analyses of the mitochondrial genome of Spuriopimpinella brachycarpa
Source: Front Plant Sci. 2024 Nov 26;15:1492723. doi: 10.3389/fpls.2024.1492723 (PMC11628310; doi:10.3389/fpls.2024.1492723)
Supplement: Supplementary Table 2 — Genes predicted in the plastome of S. brachycarpa. [file Table2.docx]

Table S2 Genes predicted in the plastome of *S. brachycarpa*

| **Category** | **Gene group** | **Gene name** |
| --- | --- | --- |
| **Photosynthesis** | Subunits of photosystem I | *psa*B, *psa*A, *psa*I, *psa*J, *psa*C |
|  | Subunits of photosystem II | *psb*A, *psb*K, *psb*I, *psb*M, *psb*D, *psb*C, *psb*Z, *psb*J, *psb*L, *psb*F, *psb*E, *psb*B, *psb*T, *psb*N, *psb*H |
|  | Subunits of NADH dehydrogenase | *ndh*J, *ndh*K, *ndh*C, *ndh*B (2), *ndh*F, *ndh*D, *ndh*E, *ndh*G, *ndh*I, *ndh*A, *ndh*H |
|  | Subunits of cytochrome b/f complex | *pet*N, *pet*A, *pet*L, *pet*G, *pet*B, *pet*D |
|  | Subunits of ATP synthase | *atp*A, *atp*F, *atp*H, *atp*I, *atp*E, *atp*B |
|  | Large subunit of rubisco | *rbc*L |
| **Self-replication** | Proteins of large ribosomal subunit | *rpl*33, *rpl*20, *rpl*36, *rpl*14, *rpl*16, *rpl*22, *rpl*2 (2), *rpl*23 (2), *rpl*32 |
|  | Proteins of small ribosomal subunit | *rps*12 (2), *rps*16, *rps*2, *rps*14, *rps*4, *rps*18, *rps*11, *rps*8, *rps*3, *rps*19, *rps*7 (2), *rps*15 |
|  | Subunits of RNA polymerase | *rpo*C2, *rpo*C1, *rpo*B, *rpo*A |
|  | Ribosomal RNAs | *rrn*16S (2), *rrn*23S (2), *rrn*4.5S (2), *rrn*5S (2) |
|  | Transfer RNAs | *trn*H-GUG, *trn*K-UUU, *trn*Q-UUG, *trn*S-GCU, *trn*G-UCC, *trn*R-UCU, *trn*C-GCA, *trn*D-GUC, *trn*Y-GUA, *trn*E-UUC, *trn*T-GGU, *trn*S-UGA, *trn*G-GCC, *trn*fM-CAU, *trn*S-GGA, *trn*T-UGU, *trn*L-UAA, *trn*F-GAA, *trn*V-UAC, *trn*M-CAU, *trn*W-CCA, *trn*P-UGG, *trn*I-CAU (2), *trn*L-CAA (2), *trn*V-GAC (2), *trn*I-GAU (2), *trn*A-UGC (2), *trn*R-ACG (2), *trn*N-GUU (2), *trn*L-UAG |
| **Other genes** | Maturase | *mat*K |
|  | Protease | *clp*P |
|  | Envelope membrane protein | *cem*A |
|  | Acetyl-CoA carboxylase | *acc*D |
|  | c-type cytochrome synthesis gene | *ccs*A |
|  | Translation initiation factor | *inf*A |

“(2)”: genes with two copies.
